# Rabies virus utilizes neuropilin 2 as an endocytic receptor to trigger TGFBR1-mediated actin polymerization

**DOI:** 10.1128/jvi.00638-25

**Published:** 2025-06-25

**Authors:** Ziruo Sun, Jinqiu Wang, Zhiyuan Wen, Lei Shuai, Wenjing Sun, Mengjie Yang, Jinyu Wang, Junyu Chen, Jinying Ge, Weiye Chen, Xijun Wang, Zhigao Bu, Jinliang Wang

**Affiliations:** 1 State Key Laboratory for Animal Disease Control, Harbin Veterinary Research Institute, Chinese Academy of Agricultural Sciences 111613 , Harbin, China; 2 Jiangsu Co-innovation Center for Prevention and Control of Important Animal Infectious Diseases and Zoonoses, Yangzhou University 38043, Yangzhou, China; University Medical Center Freiburg, Freiburg, Germany

**Keywords:** rabies virus, neuropilin 2, receptor, transforming growth factor-β receptor I, actin polymerization

## Abstract

Rabies virus (RABV), belonging to the rhabdovirus, is a typical large virus that enters cells via clathrin-mediated endocytosis (CME). RABV-containing pits are only partially clathrin-coated and require local actin polymerization for efficient internalization. This unconventional entry process suggests that a specific receptor may be required to initiate actin polymerization during RABV entry. Here, we found that RABV uses the cell membrane protein neuropilin 2 (NRP2) to initiate F-actin polymerization. NRP2 is required for RABV infection and directly interacts with RABV glycoprotein. An antibody against the ectodomain of NRP2 and the soluble ectodomain of NRP2 blocked RABV infection in cells. Expression of human NRP2 in non-susceptible DU145 cells enabled RABV infection. We further found that NRP2 interacted with transforming growth factor-β receptor I (TGFBR1), triggering TGFBR1/2-Cdc42-mediated F-actin polymerization. Vesicular stomatitis virus, another prototypical rhabdovirus, also uses a similar mechanism to enter cells. Our findings demonstrate that NRP2 is a novel receptor for RABV entry by transducing the signal of viral binding across the plasma membrane to initiate actin polymerization. NRP2 may represent one of the long-sought molecules that facilitate large pathogen cell entry via CME.

**IMPORTANCE:**

Rabies virus (RABV) enters cells via clathrin-mediated endocytosis (CME), but RABV-containing pits are only partially clathrin-coated, requiring actin polymerization for efficient entry. However, how the virus triggers the actin polymerization remains unclear. Here, we found that the cell membrane protein neuropilin 2 (NRP2) is required for RABV infection and directly interacts with RABV glycoprotein. An antibody against the ectodomain of NRP2 and the soluble ectodomain of NRP2 blocked RABV infection in cells. Expression of human NRP2 in non-susceptible DU145 cells enabled RABV infection. We further found that NRP2 interacted with transforming growth factor-β receptor I (TGFBR1), triggering TGFBR1/2-Cdc42-mediated F-actin polymerization. Vesicular stomatitis virus, another prototypical rhabdovirus, also uses a similar mechanism to enter cells. Our findings demonstrate that NRP2 is a novel receptor for RABV entry by initiating actin polymerization and may represent one of the long-sought molecules that facilitate large pathogen cell entry via CME.

## INTRODUCTION

Viruses use different pathways to enter cells (1), including clathrin-mediated endocytosis (CME) and macropinocytosis. CME generates clathrin-coated pits (CCPs) with a diameter of 60–120 nm and is usually used for cell internalization by smaller viruses, such as the influenza virus (2–4) and reovirus (5, 6). Macropinocytosis is a transient, growth factor-induced, actin-dependent endocytic process and is the predominant endocytic form for many larger viruses, such as vaccinia virus (7), Ebola virus (8), and Kaposi’s sarcoma-associated herpesvirus (KSHV) (9).

Rabies virus (RABV) is responsible for rabies, a zoonotic disease that still poses a serious threat to global public health (10). According to the WHO, about 60,000 people die because of rabies every year (11). RABV is a bullet-shaped RNA virus, belonging to the rhabdovirus. It enters host cells through receptor-dependent CME (12–15). The size of rhabdovirus is about 180 × 70 nm, much larger than the typical size of CME cargoes. Therefore, RABV must enlarge the CCPs to accommodate the viral particles, which may result in the CCPs being incompletely coated with clathrin, such that actin polymerization is required to complete the endocytosis process (12). This unconventional entry process suggests that a specific receptor may be required to initiate actin polymerization during RABV entry.

The genome of RABV encodes five major viral proteins: nucleoprotein, phosphoprotein, matrix protein, large RNA polymerase protein, and glycoprotein (G) (16). G is the only transmembrane protein on the surface of the viral envelope and is responsible for the interaction between the virion and cell membrane receptors (17). To date, four cell membrane proteins, acetylcholine receptor subunit alpha (nAchR) (18), neural cell adhesion molecule (NCAM) (19), low-affinity nerve-growth factor receptor (p75NTR) (20), and metabotropic glutamate receptor subtype 2 (mGluR2) (21), have been identified as potential receptors of RABV infection. A recent study finds that the RABV-mGluR2 complex interacts with transferrin receptor 1 (TfR1) and hijacks the endocytic signaling of TfR1 to enter cells via CME (22, 23).

In this study, we found that neuropilin 2 (NRP2) interacts with RABV G and is important for RABV infection. NRP2 triggers actin polymerization via the transforming growth factor-β receptor I/II (TGFBR1/2)-Cdc42 signaling pathway during the endocytosis of RABV. NRP2 plays a similar role during infection of vesicular stomatitis virus (VSV), another well-studied prototypical rhabdovirus. Thus, NRP2 is a novel important entry receptor for RABV and may represent a long-sought molecule that allows large pathogens to enter cells via CME.

## RESULTS

### NRP2 is an essential host factor for RABV infection

**Fig 1:**
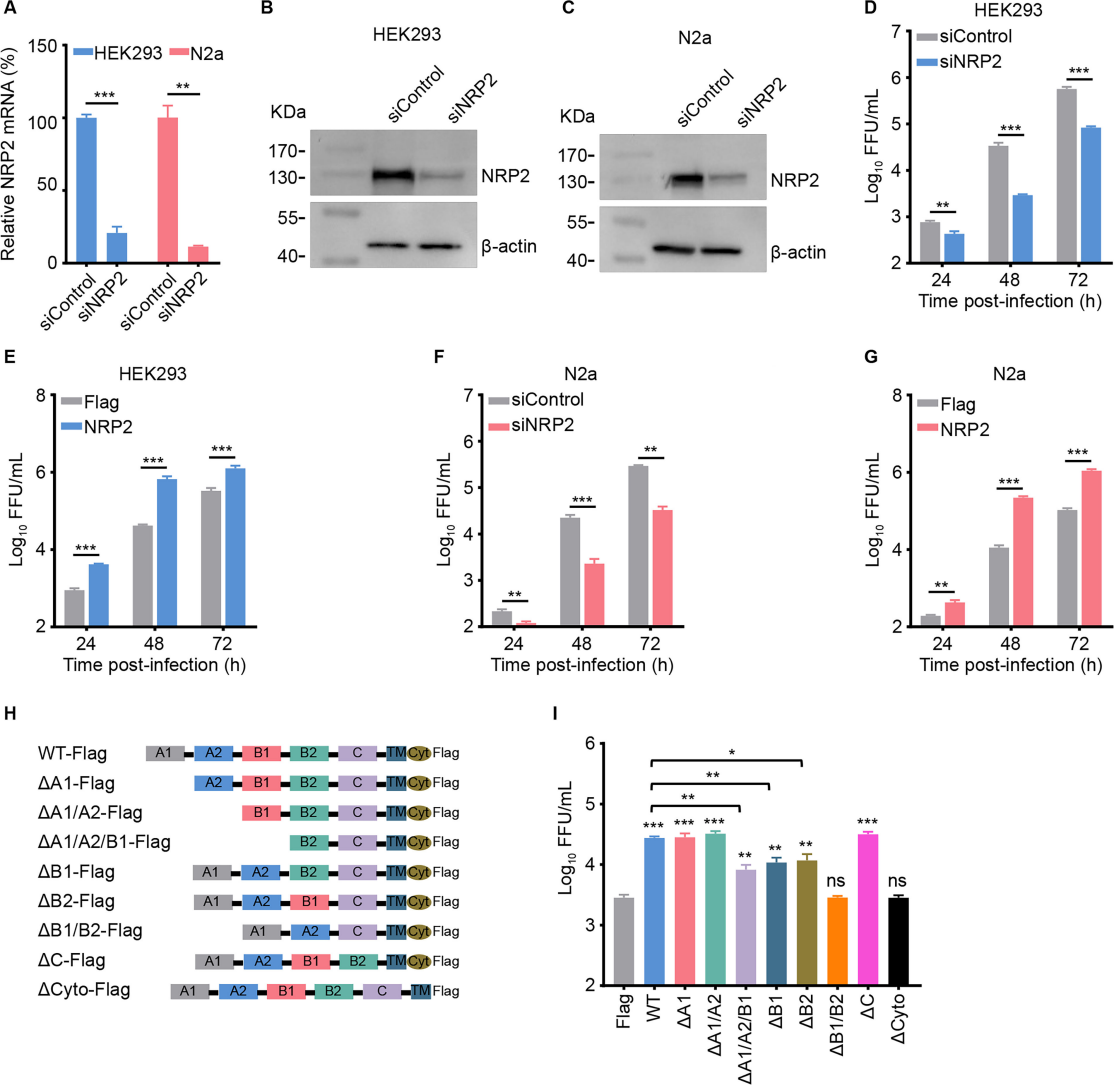
NRP2 is an essential host factor for RABV infection. (**A**) The NRP2 mRNA level in the indicated siRNA-transfected HEK293 cells or N2a cells was measured by qPCR. siNRP2, siRNAs specific for NRP2 mRNA; siControl, scrambled RNA. (**B and C**) The NRP2 protein levels in the indicated siRNA-transfected HEK293 cells (**B**) or N2a cells (**C**) were measured by Western blotting at 60 h post-transfection, respectively. siNRP2, siRNAs specific for NRP2 mRNA; siControl, scrambled RNA. (D–G) NRP2-silenced or -overexpressing HEK293 cells (**D and E**) and N2a cells (**F and G**) were infected with ERA-EGFP, respectively. Virus titers in the cell culture supernatant were determined as focus-forming units (FFU) in BSR T7/5 cells. (**H**) Schematic representation of the NRP2 mutant constructs used in the infection experiments described in panel **I**. The design of the mutant constructs was based on the reported NRP2 crystal structure. (**I**) HEK293 cells expressing vector control, Flag-tagged full-length NRP2, or the individual NRP2 mutants were infected with ERA-EGFP. Virus titers in the cell culture supernatant were determined as FFU. The data shown in panels **A**, D–G, and **I** are means ± standard deviation (SD) of three independent experiments. Statistical analysis was performed using the unpaired, two-tailed Student’s test, *P < 0.05, **P < 0.01, ***P < 0.001.

Previously, we performed a high-throughput small interfering RNA (siRNA) screen to identify host factors required for RABV infection (21), and subsequently, our analysis revealed that knockdown of NRP2 expression significantly inhibited the replication of ERA-EGFP, a recombinant RABV ERA strain that expresses enhanced green fluorescent protein. To test whether NRP2 is needed for RABV infection, we knocked down NRP2 expression by transfecting HEK293 cells and N2a cells (a mouse neuroblastoma cell line) with specific siRNA to NRP2 mRNA (siNRP2). Non-targeting scramble siRNA (siControl) was used as the negative control. Compared with siControl-transfected cells, the expression of NRP2 mRNA or protein in siNRP2-transfected cells was significantly reduced in both cell lines at 24 h or 60 h post-transfection (Fig. 1A through C). At 60 h post-transfection, HEK293 cells and N2a cells were infected with ERA-EGFP (multiplicity of infection, MOI 0.05) for 1 h at 37°C. Then the amount of virus released into the culture supernatant by the infected cells was measured by viral titration at 24 h, 48 h, and 72 h post-infection, respectively. We found that knockdown of NRP2 expression significantly decreased the viral titers at all time points tested in both cell types (Fig. 1D and F). We further tested whether NRP2 overexpression promotes RABV infection by transfecting NRP2 cDNA into HEK293 cells and N2a cells. The results showed that the viral titers were significantly higher in NRP2-overexpressing HEK293 cells and N2a cells than in mock-transfected cells at 24 h, 48 h, and 72 h post-infection (Fig. 1E and G). These results demonstrate that NRP2 plays an important role in RABV infection.

NRP2 is a type I transmembrane protein, comprising two CUB domains (A1/A2), two Factor V/VIII homology domains (B1/B2), a MAM domain (C), a single transmembrane domain, and a small cytoplasmic domain (24). We next generated a series of expression constructs deleting different domains of NRP2, as shown in Fig. 1H, to identify the key domains required for RABV infection. The different NRP2 mutant plasmids were transfected into HEK293 cells, respectively, then infected with ERA-EGFP (MOI 0.05) at 48 h post-transfection. The virus in the supernatant was titrated at 48 h post-infection. Compared with those in mock-transfected cells, the viral titers were significantly increased in cells overexpressing full NRP2 or NRP2 lacking domain A1, A2, or C, and were slightly increased in cells overexpressing NRP2 lacking domain B1 or B2. No significant changes were observed in cells overexpressing NRP2 lacking domain B1/B2 or the cytoplasmic domain (Fig. 1I). These results suggest that the domain B1/B2 and the cytoplasmic domain of NRP2 are essential for NRP2-promoted RABV infection.

### NRP2 promotes endocytosis of RABV

**Fig 2:**
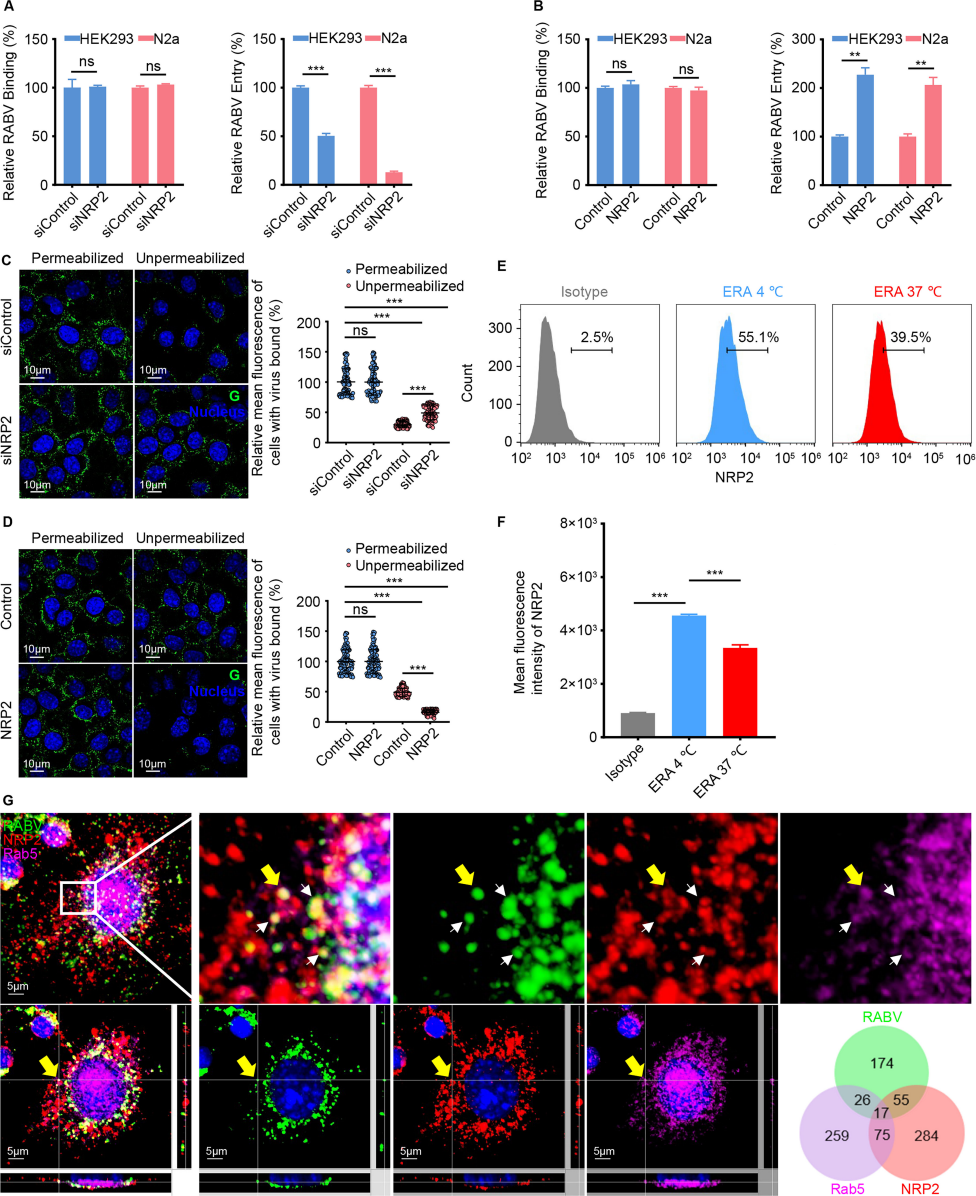
NRP2 promotes endocytosis of RABV. (**A**) Viral binding or internalization was quantified by qPCR, and the results were normalized to the respective scrambled siRNA-transfected cells. (**B**) Viral binding or internalization was quantified by qPCR, and the results were normalized to the respective vector control cells. (**C**) N2a cells were treated as described in panel **B**, except they were not treated with acid buffer/trypsin. Cell nuclei (blue) and RABV G protein (green). The fluorescence signal of RABV particles was quantified by immunofluorescence staining using ZEN software. The relative fluorescence of cell-bound RABV under permeabilized or unpermeabilized conditions was quantified by normalization to the corresponding vector control cells. The circles represent individual data points, *n* = 100. (**D**) NRP2-silenced N2a cells were treated and analyzed as described in panel **C**. The relative fluorescence of cell-bound RABV under permeabilized or unpermeabilized conditions was quantified by normalization to the corresponding scrambled siRNA-transfected cells. The circles represent individual data points, *n* = 100. (**E and F**) Cell surface expression level of NRP2 was assessed using flow cytometry after infection with ERA-EGFP at 37°C for 30 min under unpermeabilized conditions in HEK293 cells. (**G**) Multiplex immunofluorescence was performed in N2a cells. Colocalization of NRP2 (red), RABV (green), and Rab5 (purple) was observed and quantified. The arrows indicate the colocalization of NRP2 (red), RABV (green), and Rab5 (purple). One representative colocalization (yellow arrow) is shown in three dimensions. The data shown in panels A–D and **F** are means ± SD of three independent experiments. Statistical analysis was performed using the unpaired, two-tailed Student’s test, ns, not significant, **P < 0.01, ***P < 0.001.

NRP2 is abundant in the central nervous system (CNS) (25–28). Since RABV is a typical neurotropic virus (29), we speculated that NRP2 may serve as a receptor for RABV entry into cells. To test this hypothesis, we performed RNAi assays to determine whether NRP2 affected RABV entry. NRP2-silenced or NRP2-overexpressing HEK293 cells and/or N2a cells were incubated with ERA-EGFP (MOI 10) at 4°C for 1 h and shifted to 37°C for 2 h to allow the entry of bound viruses. Cells were washed using trypsin before lysis for qPCR of RABV. Cells were also directly fixed for immunofluorescence staining with an antibody against RABV G to visualize the viral particles under permeabilized and unpermeabilized conditions. The results of qPCR revealed that the viruses bound on the NRP2-silenced cells were comparable to those on the siControl-transfected cells; however, the amount of virus that entered NRP2-silenced cells was significantly decreased compared with that for siControl-transfected cells (Fig. 2A). NRP2 overexpression had no effect on RABV-binding cells while significantly promoting the endocytosis of RABV (Fig. 2B). These results indicate that NRP2 is involved in the endocytosis of RABV but not in RABV binding.

In immunofluorescence analyses, the fluorescence intensity under permeabilized conditions is a measure of the total number of viral particles, whereas under unpermeabilized conditions, it indicates that the viral particles were unable to enter the cells. Under permeabilized conditions, the fluorescence intensity in NRP2-silenced or NRP2-overexpressed N2a cells was comparable to that of their respective control cells, indicating that NRP2 does not affect RABV binding (Fig. 2C and D). However, under unpermeabilized conditions, the fluorescence intensity in NRP2-silenced N2a cells was significantly higher than that of control cells, whereas the fluorescence intensity in NRP2-overexpressed N2a cells was significantly lower than that of control cells, indicating that NRP2 is required for the endocytosis of RABV (Fig. 2C and D).

We then investigated whether NRP2 is internalized with RABV. HEK293 cells were incubated with ERA-EGFP (MOI 10) at 4°C for 1 h and shifted to 37°C for 1 h. Cells were harvested immediately before and after the 37°C incubation. The cells were stained with an antibody against NRP2 under unpermeabilized conditions to determine the molecular composition of the cell membrane by flow cytometry. The fluorescence intensity of NRP2 in the cells after the 37°C incubation was significantly lower than that before the 37°C incubation (Fig. 2E and F). These results indicate that NRP2 on the cell surface is internalized together with RABV during endocytosis. Because it has been indicated that RABV is transported to the early endosomes after internalization (30), we next examined the subcellular localization of RABV particles, NRP2, and the early endosome marker Rab5 using multiplex immunofluorescence staining in ERA_G/Flag_-infected N2a cells. We found that RABV particles, NRP2, and Rab5 colocalized in infected cells (Fig. 2G), indicating that RABV and NRP2 internalize together.

### NRP2 interacts with RABV G

**Fig 3:**
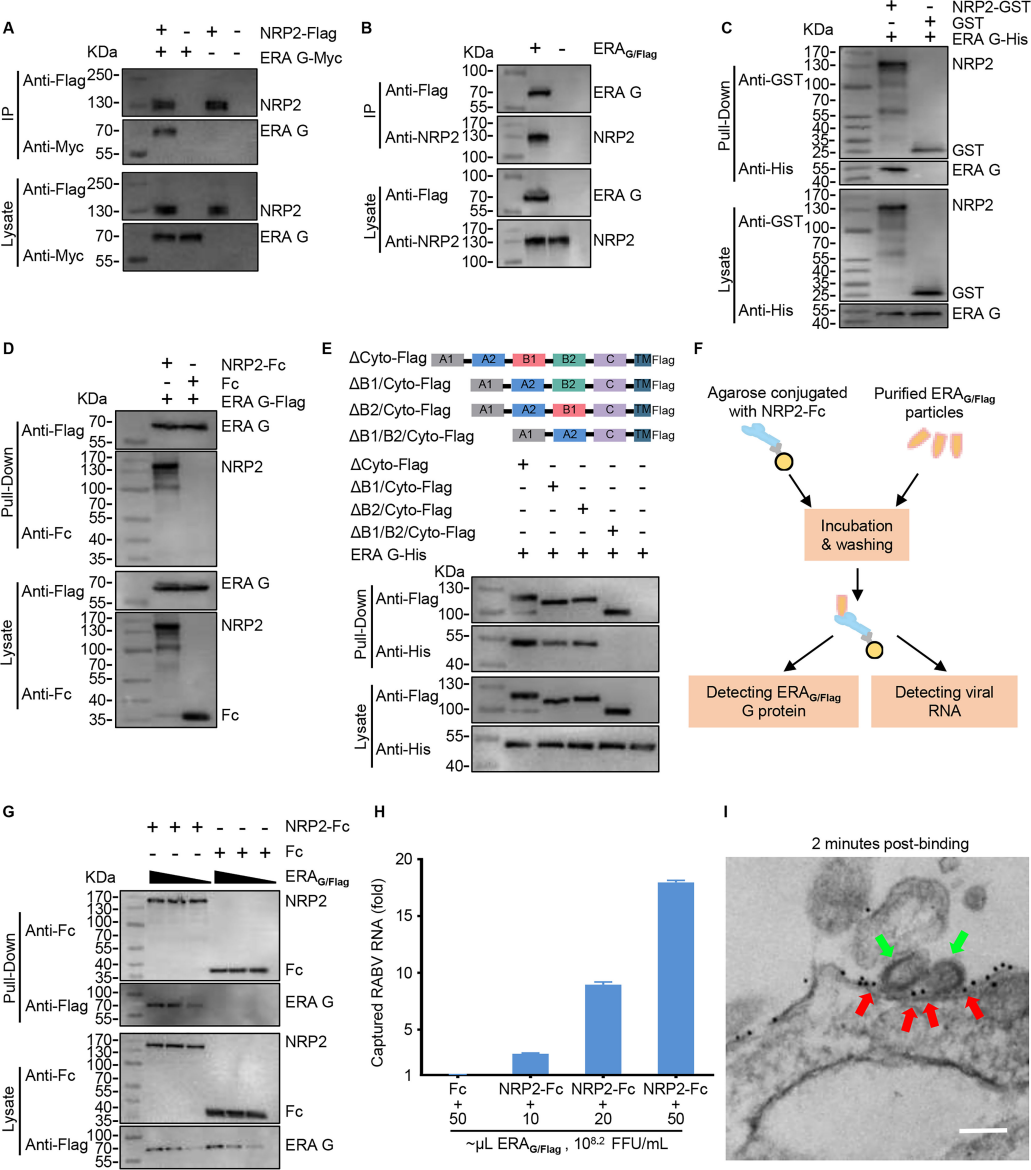
NRP2 interacts with RABV G. (**A**) HEK293 cells were co-transfected with NRP2-Flag and ERA G-Myc, and then subjected to immunoprecipitation (IP) using anti-Flag agarose beads. Representative western blots of whole-cell lysates and eluates after IP are shown. (**B**) HEK293 cells were infected with ERAG/Flag (MOI 0.05) and then subjected to IP using anti-Flag agarose beads at 48 h post-infection. (**C**) NRP2-GST was pooled with ERA G-His and then pulled down using anti-GST beads. The GST protein was used as the negative control. (**D**) NRP2-Fc was pooled with ERA G-Flag and then pulled down using anti-Flag agarose beads. The Fc protein was used as the negative control. (**E**) The lysates from ΔCyto-Flag-, ΔB1/Cyto-Flag-, ΔB2/Cyto-Flag-, or ΔB1/B2/Cyto-Flag-transfected HEK293 cells were pooled with purified G-His protein and then pulled down using anti-Flag agarose beads. (**F**) Schematic representation of the detection of the interaction between NRP2 and RABV particles. (**G**) NRP2-Fc was pooled with different amounts of ERA_G/Flag_ particles (10, 20, or 50 µL, FFU/mL = 10^8.2^) and then pulled down using protein G beads. The Fc protein was used as the negative control. (**H**) Captured RABV particles were quantified in NRP2-Fc-conjugated protein G beads by qPCR, and the results were normalized to the Fc control group. (**I**) NRP2 molecules were detected on the cell membrane where RABV attached by the use of immunoelectron microscopy in NRP2-overexpressing N2a cells. Green arrow, RABV; red arrow, NRP2. Scale bar, 200 nm.

A direct interaction between NRP2 and RABV G is a prerequisite for NRP2 being a RABV receptor. We next tested the interaction of RABV G and NRP2 by performing co-immunoprecipitation assays using Flag-tagged NRP2 protein (NRP2-Flag) and Myc-tagged RABV G derived from the cell-adapted strain ERA, the mouse-adapted strain CVS-24, and the street virus GX/09 in plasmid-transfected HEK293 cells, respectively. All three G proteins interacted with NRP2 (Fig. 3A; Fig. S1A). The interaction of endogenous NRP2 and ERA G-Flag derived from HEK293 cells infected with ERA_G/Flag_, a recombinant RABV ERA strain expressing G fused to a Flag-tag at the C terminus, was confirmed by co-immunoprecipitation assays (Fig. 3B). We then performed a pull-down assay. The purified recombinant GST-tagged NRP2 ectodomain (NRP2-GST) was pooled with the purified recombinant His-tagged RABV G ectodomain derived from the ERA strain (ERA G-His). ERA G-His was successfully pulled down by NRP2-GST (Fig. 3C). Similarly, CVS-24 G, GX/09 G, and another member of the Rhabdoviridae family, West Caucasian bat virus, WCBV G, were successfully pulled down by NRP2-GST (Fig. S1B). We also expressed and purified a recombinant Fc-tagged NRP2 ectodomain (NRP2-Fc) in HEK293F cells, and then performed a pull-down assay in HEK293 cells that were transfected with Flag-tagged ERA G. The results showed that NRP2-Fc was successfully pulled down by ERA G-Flag (Fig. 3D). NRP2 lacking domain B1/B2 almost completely failed to pull down ERA G-His (Fig. 3E), suggesting that domain B1/B2 of NRP2 is essential for the interaction with RABV G.

We further investigated the interaction of NRP2 and RABV using NRP2-Fc and purified RABV particles. We pooled the NRP2-Fc with different amounts of RABV particles and subsequently captured them with protein G agarose. The interaction of RABV particles with NRP2 was determined by monitoring captured RABV G protein and RABV virion-associated RNA, respectively (Fig. 3F). Compared with the Fc protein control, RABV particles were efficiently captured by the NRP2-Fc through protein G agarose. The amount of RABV particles captured by NRP2-Fc increased in a dose-dependent manner with an increase in the amount of RABV particles (Fig. 3G and H). Moreover, the colocalization of NRP2 and RABV particles in HEK293 cells was further confirmed by immunoelectron microscopy (Fig. 3I). These results demonstrate that NRP2 directly interacts with RABV G.

### NRP2 ectodomain soluble protein and NRP2 antibody inhibit RABV infection of cells

**Fig 4:**
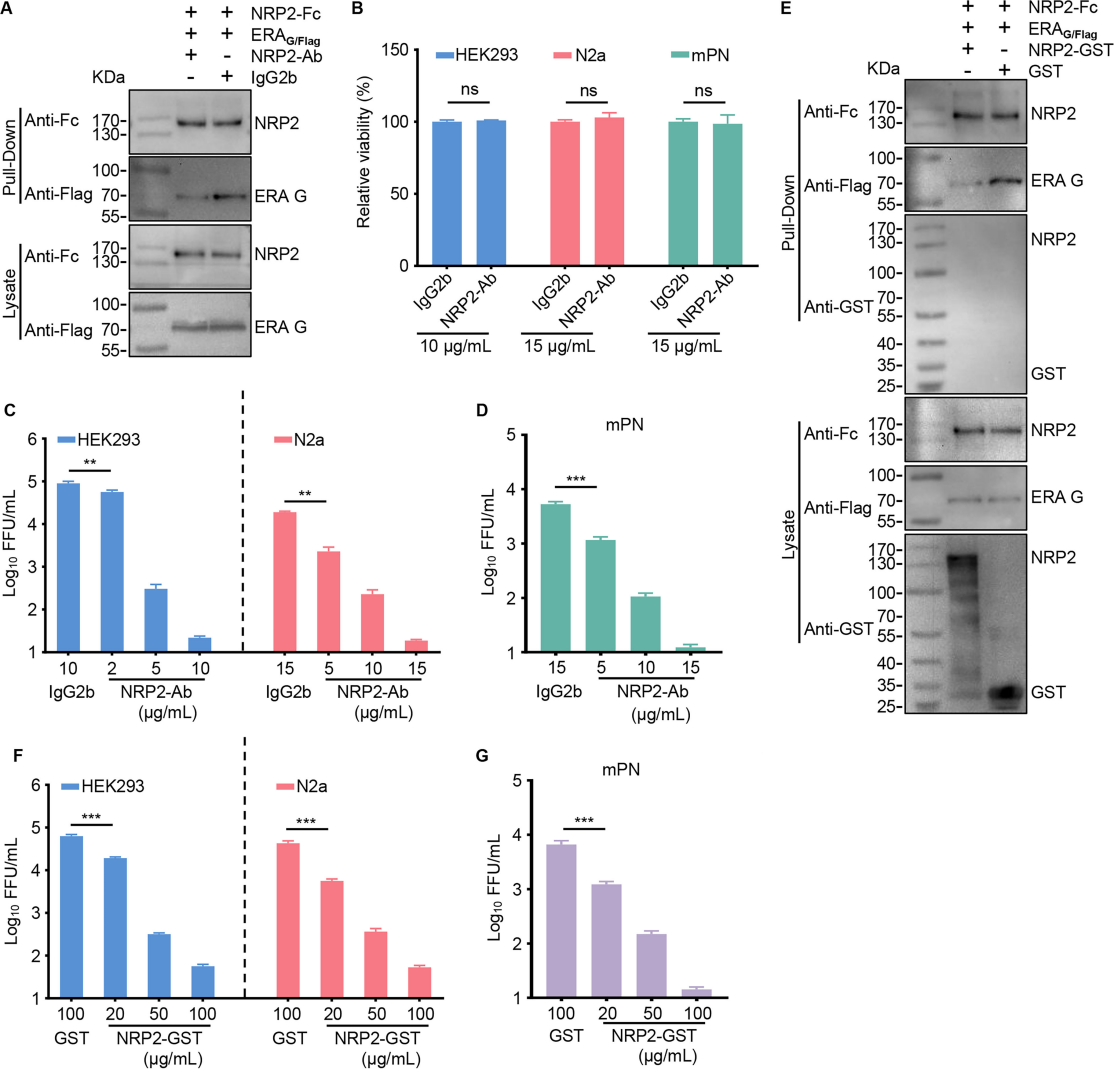
Both NRP2 antibody and NRP2 ectodomain soluble protein inhibit RABV infection of cells. (**A**) NRP2-Fc was incubated with NRP2-Ab or IgG2b for 1 h at 4°C, respectively. Then the mixture was incubated with purified ERA_G/Flag_ particles after being conjugated with protein G agarose beads. The samples were subjected to SDS-PAGE and western blot analysis. (**B**) HEK293 cells, N2a cells, or mPN cells were treated with NRP2-Ab or IgG2b for 48 h. Cell viability was determined using a Cell Titer Glo kit. (**C and D**) HEK293 cells, N2a cells, or mPN cells were treated with NRP2-Ab at different concentrations or IgG2b (10 µg/mL for HEK293 cells; 15 µg/mL for N2a cells and mPN cells) for 1 h at 4°C, and then infected with ERA-EGFP. Virus titers in the cell culture supernatant were determined as FFU at 48 h p.i. (**E**) Purified ERA_G/Flag_ particles were pooled with NRP2-GST or GST for 1 h at 4°C, respectively. Then they were mixed with NRP2-Fc-conjugated protein G agarose beads. The samples were subjected to SDS-PAGE and western blot analysis. (**F and G**) ERA-EGFP was pooled with different concentrations of NRP2-GST or GST (100 µg/mL), and then HEK293 cells, N2a cells, and mPN cells were infected with the different mixtures, respectively. Virus titers in the cell culture supernatant were determined as FFU at 48 h p.i. The data shown in panels B–D, F, and G are means ± SD of three independent experiments. Statistical analysis was performed using the unpaired, two-tailed Student’s test, ns, not significant, **P < 0.01, ***P < 0.001.

We then performed antibody blocking assays and soluble NRP2 protein neutralization assays to investigate whether the interaction between NRP2 and G is important for RABV infection as described previously (21). A monoclonal antibody against the ectodomain of NRP2 (NRP2-Ab) was used for the antibody blocking assays, and the isotype control antibody (IgG2b) was used as a negative control. We confirmed that the NRP2-Ab can weaken the interaction between NRP2 and RABV particles using a protein interaction competition assay (Fig. 4A). HEK293 cells and N2a cells were treated with the NRP2 antibody at the indicated concentrations for 1 h at 4°C, and then incubated with ERA-EGFP (MOI 0.05). Infectious titers in the supernatant were detected by virus titration at 48 h post-infection. Cell viability was not affected by NRP2-Ab (Fig. 4B). NRP2-Ab efficiently inhibited RABV infection in HEK293 cells and N2a cells in a dose-dependent manner (Fig. 4C). This finding was confirmed using mouse primary neuronal (mPN) cells. NRP2-Ab was not cytotoxic at the concentration used in assays and significantly decreased RABV infection in mPN cells (Fig. 4B and D).

NRP2-GST was used for inhibition assays, and purified GST was used as a negative control. The result from the protein interaction competition assay showed that NRP2-GST can disrupt the interaction between NRP2 and RABV particles (Fig. 4E). ERA-EGFP (MOI 0.05) was pooled with different concentrations of NRP2-GST for 1 h at 4°C, and then HEK293 cells, N2a cells, and mPN cells were incubated with the mixtures for 1 h at 37°C, respectively. The infectious titers in the supernatants of the infected cells were detected at 48 h post-infection. NRP2-GST inhibited ERA-EGFP infection in a dose-dependent manner in all three cell types (Fig. 4F and G). These results demonstrate that the interaction between NRP2 and G is important for RABV infection.

### NRP2 rescues RABV infection in non-susceptible cells

**Fig 5:**
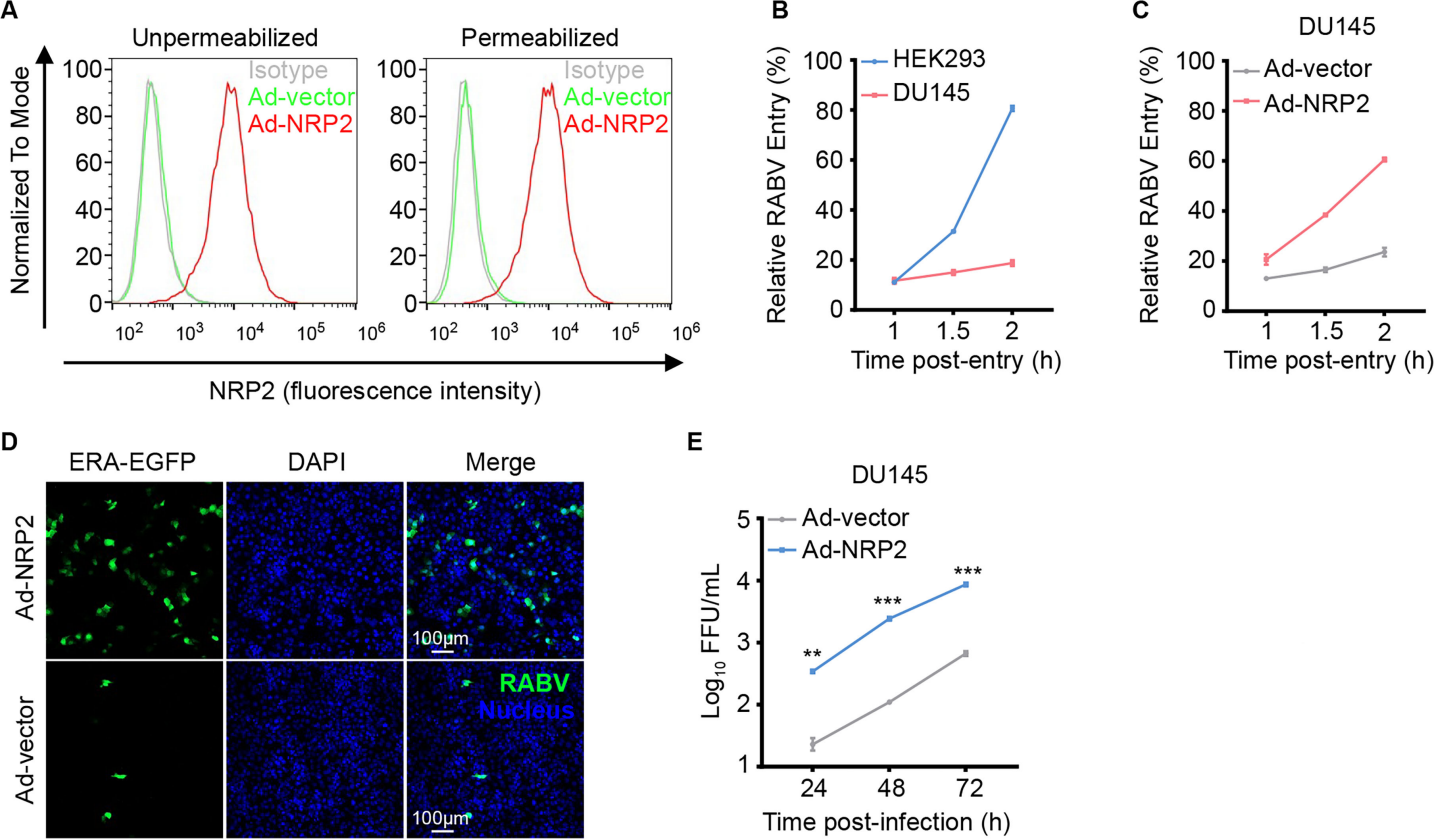
NRP2 rescues RABV infection in non-susceptible cells. (**A**) The expression of NRP2 under unpermeabilized or permeabilized conditions was detected by flow cytometry in DU145 cells transduced with Ad-vector or Ad-NRP2. (**B**) The kinetics of RABV that entered HEK293 cells or DU145 cells was measured by qPCR at the indicated times post-entry. (**C**) The kinetics of RABV that entered Ad-vector- or Ad-NRP2-transduced DU145 cells was measured by qPCR at the indicated times post-entry. (**D and E**) DU145 cells transduced with Ad-vector or Ad-NRP2 were infected with ERA-EGFP. EGFP-positive cells were visualized by fluorescence microscopy (**D**), and the viral titers in the cell culture supernatant were determined as FFU (**E**). The data shown in panel **E** are means ± SD of three independent experiments. Statistical analysis was performed using the unpaired, two-tailed Student’s test, **P < 0.01, ***P < 0.001.

A previous study suggests that DU145, a prostate carcinoma cell line, does not express NRP2 (31). We confirmed that DU145 cells did not express NRP2 by flow cytometry analysis (Fig. 5A) and found that RABV endocytosis was highly limited in DU145 cells (Fig. 5B). To test whether NRP2 rescues the infection of RABV in DU145 cells, we transduced DU145 cells with a recombinant human adenovirus 5 (Ad) vector expressing the NRP2 cDNA and then assessed RABV endocytosis and infection. The expression of NRP2 on the cell surface of DU145 cells was confirmed by flow cytometry analysis (Fig. 5A). We found that virus entry and infectivity were significantly increased in NRP2-overexpressing DU145 cells (Fig. 5C through E), indicating that NRP2 confers RABV infectivity to DU145 cells.

### TGFBR1/2 is involved in NRP2-mediated endocytosis of RABV

**Fig 6:**
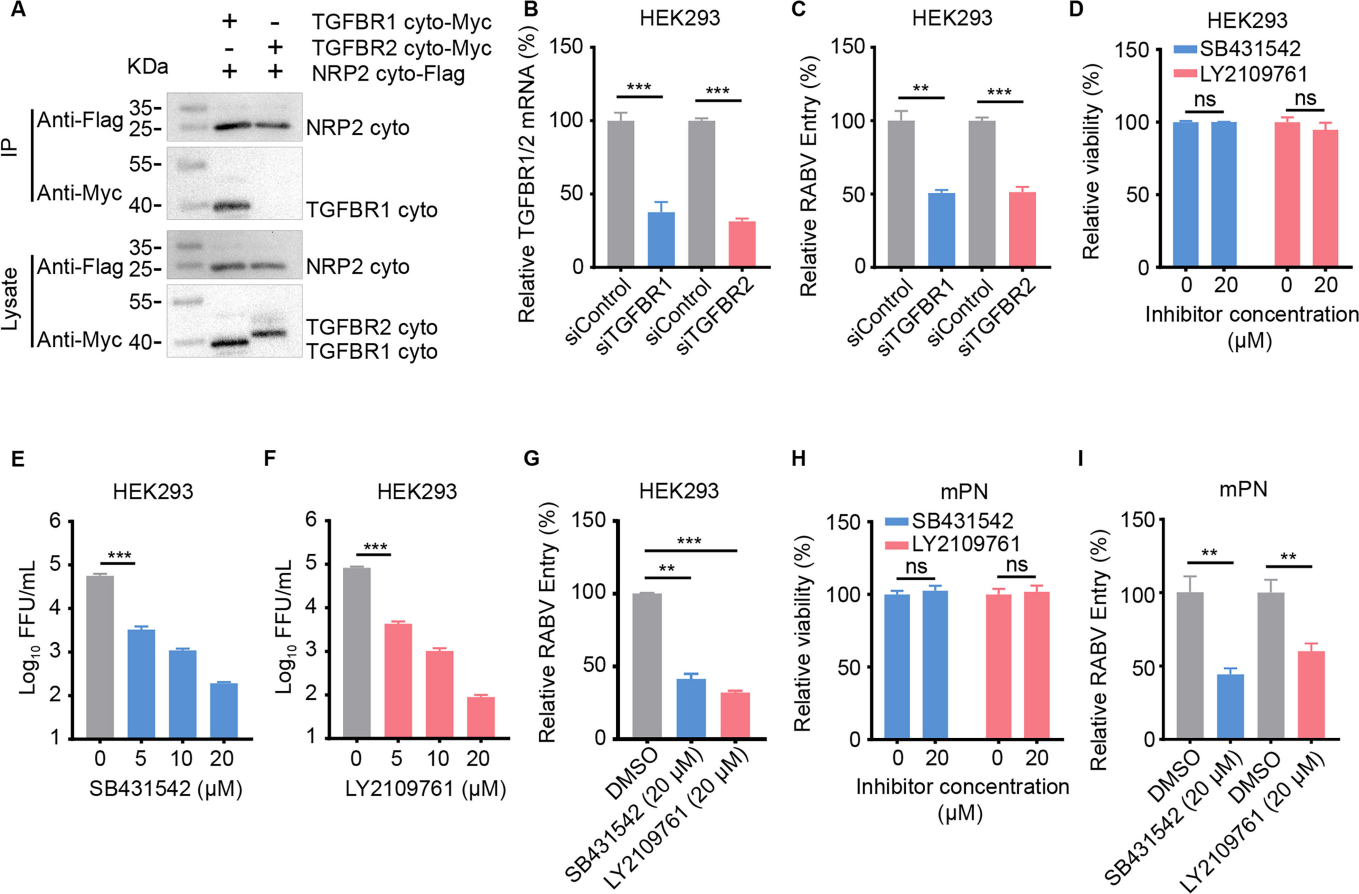
TGFBR1/2 is involved in NRP2-mediated endocytosis of RABV. (**A**) HEK293 cells were co-transfected with expression plasmids for the cytoplasmic domains of NRP2 and TGFBR1 or TGFBR2, and then subjected to IP using anti-Flag agarose beads. Representative western blots of whole-cell lysates and eluates after IP are shown. (**B**) The mRNA levels of TGFBR1 and TGFBR2 in the indicated siRNA-transfected HEK293 cells were measured by qPCR, respectively. siTGFBR1, siRNA specific for TGFBR1 mRNA; siTGFBR2, siRNA specific for TGFBR2 mRNA. (**C**) RABV internalization was quantified in TGFBR1- or TGFBR2-silenced HEK293 cells by qPCR, and the results were normalized to the respective scrambled siRNA-transfected cells. (**D**) HEK293 cells were treated with SB431542 or LY2109761 at the indicated concentrations for 48 h at 37°C, and then cell viability was determined using the Cell Titer Glo kit. (**E and F**) HEK293 cells treated with SB431542 (**E**) or LY2109761 (**F**) at the indicated concentrations were infected with ERA-EGFP, and virus titers in the cell culture supernatant were determined as FFU. (**G**) RABV internalization was quantified in SB431542- or LY2109761-treated HEK293 cells by qPCR, and the results were normalized to DMSO-treated cells. (**H**) The cell viability of mPN cells was determined as described in panel **D**. (**I**) RABV internalization was quantified in SB431542- or LY2109761-treated mPN cells by qPCR, and the results were normalized to DMSO-treated cells. The data shown in panels B and D to I are means ± SD of three independent experiments. Statistical analysis was performed using the unpaired, two-tailed Student’s test, ns, not significant, **P < 0.01, ***P < 0.001.

NRP2 binds to a broad repertoire of ligands and transduces intercellular signals via interactions with different adaptor proteins (32–35). Our data showed that the cytoplasmic domain of NRP2 is required for RABV infection (Fig. 1G), indicating that a specific signaling pathway may be activated upon RABV binding to NRP2. Previous studies have reported that NRP1 can bind to transforming growth factor-β receptor II (TGFBR2) through their respective cytoplasmic domains (36). Given the structural similarity between NRP1 and NRP2, we hypothesized that the cytoplasmic domain of NRP2 may also interact with TGFBR2. Unexpectedly, the result of the co-immunoprecipitation assay showed that the cytoplasmic domain of TGFBR2 did not interact with the cytoplasmic domain of NRP2 (Fig. 6A). TGFBR2 can form a heteromeric protein kinase receptor complex with transforming growth factor-β receptor I (TGFBR1) to mediate TGF-β signaling (37). Therefore, we speculate that NRP2 interacts with TGFBR1 through their respective cytoplasmic domains, and co-immunoprecipitation assay results confirmed this hypothesis (Fig. 6A).

We next performed RNAi assays in HEK293 cells and found that silencing TGFBR1 or TGFBR2 significantly decreased the endocytosis of RABV, suggesting that TGFBR1 and TGFBR2 are involved in RABV entry (Fig. 6B and C). To confirm the effect of the TGFBR1/2 complex on the endocytosis of RABV, HEK293 cells were pre-incubated with the inhibitors of TGFBR1 (SB431542) and TGFBR2 (LY2109761) at the indicated concentration for 1 h at 37°C, respectively, and then infected with ERA-EGFP (MOI 0.05). The infectious titers in the supernatants of the infected cells were evaluated at 48 h post-infection. SB431542 or LY2109761 treatment had no effect on cell viability at a concentration of 20 µM in HEK293 cells and significantly inhibited RABV infection and endocytosis (Fig. 6D through G). Moreover, both SB431542 and LY2109761 significantly inhibited the endocytosis of RABV in mPN cells (Fig. 6H and I). These results indicate that NRP2 promotes endocytosis of RABV through interacting with the TGFBR1/2 complex.

### Cdc42-dependent actin polymerization is required for NRP2-mediated RABV entry

**Fig 7:**
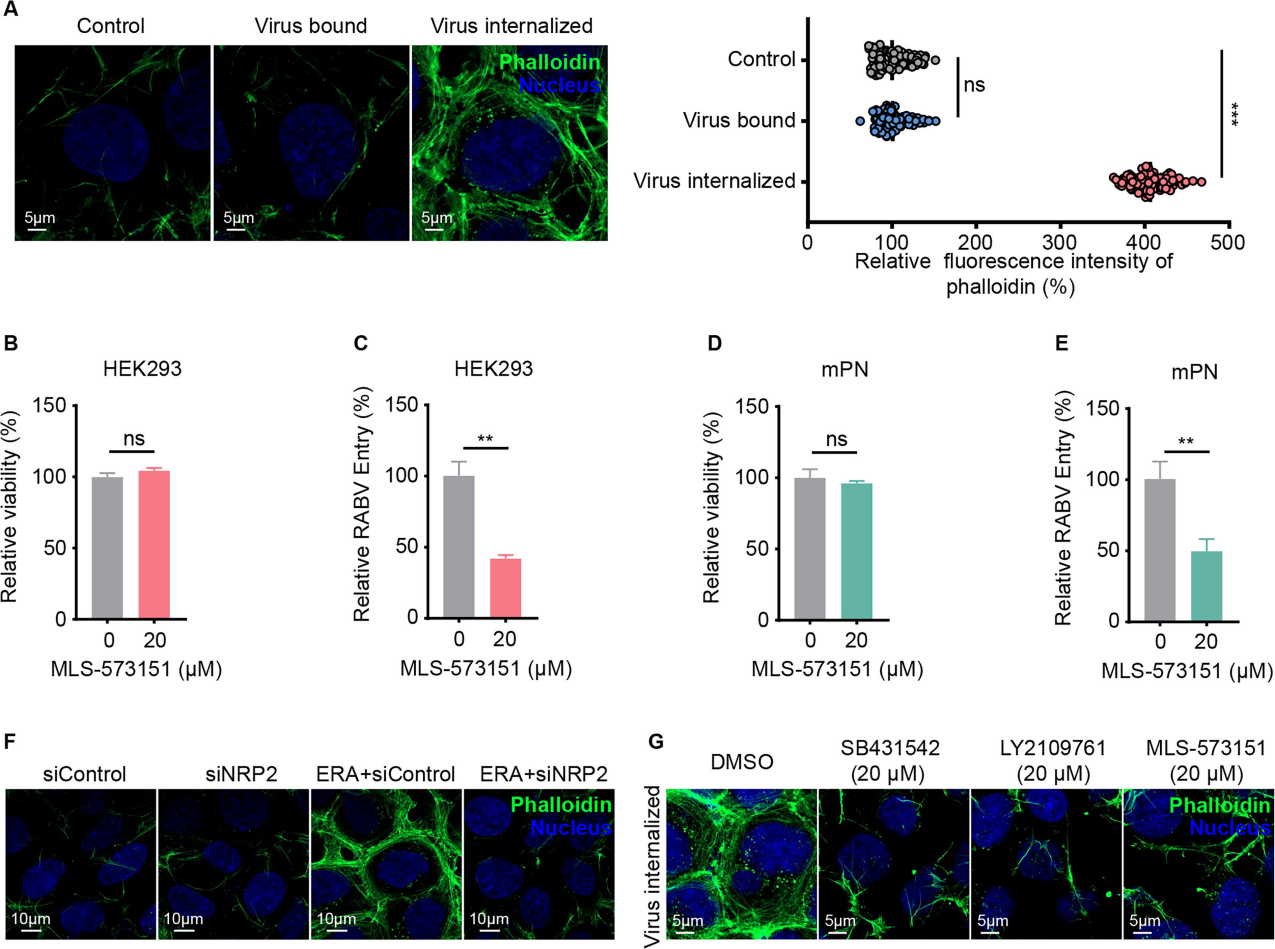
TGFBR1/2-activated and Cdc42-dependent actin polymerization is required for NRP2-mediated RABV entry. (**A**) The F-actin was visualized with Phalloidin-iFluor 488 reagent in HEK293 control cells, HEK293 cells following RABV binding or internalization. Cell nuclei (blue) and phalloidin (green). The fluorescence intensity of phalloidin was quantified using ZEN software. The relative fluorescence of phalloidin in RABV-bound or internalized HEK293 cells was quantified by normalization to control cells. The circles represent individual data points, *n* = 100. (**B and D**) HEK293 cells (**B**) or mPN cells (**D**) were treated with MLS-573151 at the indicated concentration for 48 h at 37°C, and then cell viability was determined using the Cell Titer Glo kit. (**C and E**) RABV internalization was quantified in MLS-573151-treated HEK293 cells (**C**) or mPN cells (**E**) by qPCR, and the results were normalized to DMSO-treated cells. (**F**) The F-actin was visualized with Phalloidin-iFluor 488 reagent in ERA or mock-infected siNRP2 or siControl-transfected HEK293 cells. Cell nuclei (blue) and phalloidin (green). (**G**) HEK293 cells were pretreated with DMSO, SB431542, LY2109761, or MLS-573151, then treated as described in panel **F**. Cell nuclei (blue) and phalloidin (green). The data shown in panels A–E are means ± SD of three independent experiments. Statistical analysis was performed using the unpaired, two-tailed Student’s test, ns, not significant, **P < 0.01.

RABV enters cells in CCPs with an incomplete clathrin coat, which means actin polymerization is required to complete the endocytosis process (12). To determine whether actin polymerization is induced by RABV binding or internalization, we evaluated actin polymerization in HEK293 cells following RABV binding or internalization. After RABV binding, HEK293 cells were shifted to 37°C for 30 minutes to allow RABV internalization. The cells were fixed and incubated with the F-actin dye phalloidin-iFluor 488 for 1 h before fluorescence observation. The RABV mock-incubated HEK293 cells were used as the negative control. The fluorescence intensity of phalloidin in RABV-bound HEK293 cells was comparable to that in control HEK293 cells, while it was lower than that in RABV-internalized HEK293 cells, indicating that actin polymerization is induced by RABV internalization (Fig. 7A). The TGFBR1/2 complex plays an important role in rapid actin reorganization via the activation of the Rho-like GTPases Rac1 and Cdc42 (38). To test whether the TGFBR1/2 complex promotes RABV entry by regulating actin polymerization, we first explored the effect of Rac1 and Cdc42 on RABV endocytosis in HEK293 cells. We found that Rac1 silencing or Rac1 inhibitor (EHT 1864) treatment did not significantly affect the endocytosis of RABV (Fig. S2A through D), whereas Cdc42 inhibitor (MLS-573151) treatment significantly decreased the endocytosis of RABV in HEK293 cells (Fig. 7B and C). These results indicate that Cdc42, but not Rac1, is required for RABV entry. We also confirmed this result in mPN cells (Fig. 7D and E).

We then evaluated the effect of NRP2 on RABV-induced actin polymerization by fluorescence microscopy. siControl- or siNRP2-transfected HEK293 cells were incubated with or without ERA (MOI 10) at 37°C for 30 min. Cells were then fixed and incubated with the phalloidin-iFluor 488 for 1 h before fluorescence observation. The fluorescence intensity of phalloidin in RABV mock-infected siNRP2-transfected HEK293 cells was comparable to that in siControl-transfected HEK293 cells, indicating that NRP2 knockdown does not affect actin polymerization in the absence of RABV (Fig. 7F). The fluorescence intensity of phalloidin in RABV-infected siControl-transfected cells was significantly higher than that in RABV-infected NRP2-knockdown cells (Fig. 7F), indicating that NRP2 affects RABV-induced F-actin polymerization. To evaluate whether TGFBR1/2 and Cdc42 affect RABV-induced actin polymerization, DMSO-, SB431542-, LY2109761-, or MLS-573151-treated HEK293 cells were incubated with ERA (MOI 10) at 37°C for 30 min. The fluorescence intensity of phalloidin in DMSO-treated cells was significantly higher than that in SB431542-, LY2109761-, or MLS-573151-treated cells, indicating that treatment with SB431542, LY2109761, or MLS-573151 of HEK293 cells significantly inhibits F-actin polymerization induced by RABV internalization (Fig. 7G). Taken together, these results suggest that NRP2 promotes RABV infection through TGFBR1/2-Cdc42-mediated actin polymerization.

### NRP2 is involved in the internalization of VSV but not influenza virus

**Fig 8:**
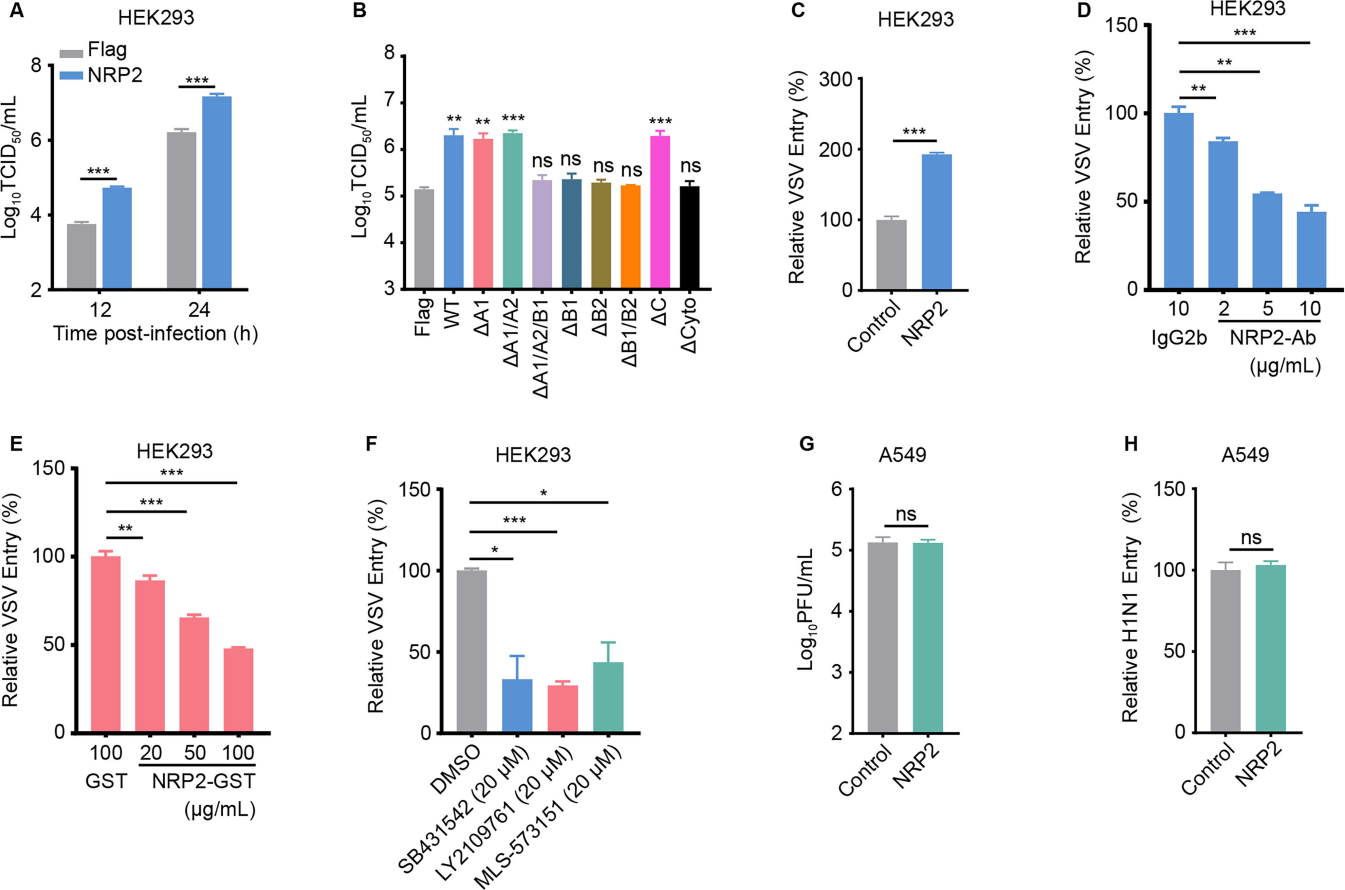
NRP2 is involved in the internalization of VSV but not influenza virus. (**A**) NRP2-overexpressing HEK293 cells were infected with VSV. Virus titers in the cell culture supernatant were determined as 50% tissue culture infectious doses (TCID_50_) in BSR T7/5 cells. (**B**) HEK293 cells expressing vector control, Flag-tagged full-length NRP2, or the individual NRP2 mutants were infected with VSV. Virus titers in the cell culture supernatant were determined as TCID_50_. (**C**) VSV internalization was quantified by qPCR, and the results were normalized to the vector control cells. (**D**) VSV internalization was quantified in NRP2-Ab-treated HEK293 cells by qPCR, and the results were normalized to IgG2b-treated cells. (**E**) VSV was pooled with different concentrations of NRP2-GST or GST (100 µg/mL), and HEK293 cells were infected with the different mixtures. Then, VSV internalization was performed and quantified. The results were normalized to the GST negative control. (**F**) VSV internalization was quantified in SB431542-, LY2109761-, or MLS-573151-treated HEK293 cells by qPCR, and the results were normalized to DMSO-treated cells. (**G**) NRP2-overexpressing A549 cells were infected with H1N1. Virus titers in the cell culture supernatant were determined as plaque-forming units (PFU) in MDCK cells. (**H**) H1N1 internalization was quantified by qPCR, and the results were normalized to the vector control cells. The data shown in panels A–H are means ± SD of three independent experiments. Statistical analysis was performed using the unpaired, two-tailed Student’s test, ns, not significant, *P < 0.05, **P < 0.01, ***P < 0.001.

VSV is another well-studied rhabdovirus and enters cells similarly to RABV (39, 40). We therefore investigated whether NRP2 is involved in the internalization of VSV. We found that overexpression of NRP2 significantly promoted the infection and internalization of VSV in HEK293 cells, and that the B1/B2 and cytoplasmic domains of NRP2 were essential for NRP2-promoted VSV infection (Fig. 8A through C). Both NRP2-Ab and NRP2-GST efficiently inhibited VSV internalization in HEK293 cells in a dose-dependent manner (Fig. 8D and E). Importantly, TGFBR1/2 and Cdc42 were also involved in the internalization of VSV (Fig. 8F). These results indicate that NRP2-mediated actin polymerization is required for the internalization of VSV. By contrast, we found that NRP2 was not required for influenza virus infection and internalization in A549 cells (Fig. 8G and H), indicating that NRP2-mediated viral CME is cargo specific.

## DISCUSSION

RABV-containing pits are only partially clathrin-coated and require local actin polymerization for efficient internalization (12). To date, four potential receptors have been identified for RABV infection (18–21), but none of them have been shown to initiate actin polymerization, suggesting that a specific receptor may be required to initiate actin polymerization during RABV entry. In this study, we found that NRP2 interacts directly with RABV G and serves as an endocytic receptor for RABV. Following its interaction with RABV G, NRP2 activates the TGFBR1/2-Cdc42 signaling pathway via an interaction with TGFBR1, thereby regulating F-actin polymerization during RABV entry. NRP2 appears to be the long-sought molecule that transduces the signal of RABV binding across the plasma membrane to initiate actin polymerization. The ectopic expression of NRP2 in DU145 cells, which are characterized by the absence of endogenous NRP2 and are non-susceptible to RABV infection (31), restores RABV infection. Interestingly, actin polymerization during RABV entry is induced by RABV internalization but not binding. Our previous studies have demonstrated that the endocytic receptor mGluR2 hijacks the internalization signal of TfR1 to mediate the CME of RABV (21, 23). These findings indicate that RABV requires distinct endocytic receptors collaborating to complete the internalization process, some to initiate the CME pathway and others to trigger actin polymerization.

NRP2 is a transmembrane receptor and has an important role in axon guidance and lymphangiogenesis (41, 42). The extracellular regions of NRP2 comprise the A1/A2 domain, B1/B2 domain, and C domain. Previous studies have suggested that the A1/A2 and B2 domains of NRP2 can bind to the human cytomegalovirus Pentamer (43); the A1 domain of NRP2 is sufficient to mediate Lujo virus entry (44). Our results demonstrated that NRP2 interacts with RABV G via the B1/B2 domain. To our knowledge, there have been no previous reports of NRP2 interacting with viral proteins through this domain. The B1/B2 domain mediates binding to the canonical carboxyl-terminal arginine (CendR) motif (R/KXXR/K) on its ligands (45). NRP2 binds to the CendR motif and initiates subsequent internalization. Several viruses can use their CendR motif to bind to neuropilin 1 (NRP1), a transmembrane receptor that has a similar domain structure to that of NRP2 (33, 46). Such viruses include severe acute respiratory syndrome coronavirus 2 (47, 48), human T-cell lymphotropic virus type 1 (49), and Epstein-Barr virus (50). There are eight potential CendR motifs in the ectodomain of RABV G, and we found that the elimination of CendR motifs does not affect the interaction of RABV G with NRP2 (Fig. S3A and B), suggesting that RABV binding to NRP2 is independent of its CendR motifs. In the future, it would be valuable to resolve the structure of the NRP2-RABV G complex to explore the key regions of RABV G that interact with NRP2.

Actin polymerization is crucial for membrane bending in the formation of CCPs (51). However, the actin polymerization induced by RABV is different from that of CME, because NRP2 is dispensable for influenza virus entry, a general CME cargo. A previous study showed that KSHV, a large DNA virus that enters cells via macropinocytosis, uses NRP1 to activate the TGFBR1/2-Cdc42/Rac1 signaling pathway via an interaction with TGFBR2 to regulate F-actin polymerization (36). Our study revealed that NRP2 activates TGFBR1/2-Cdc42 signaling by interacting with TGFBR1 to regulate F-actin polymerization during RABV entry. Therefore, RABV-induced actin polymerization is somewhat similar to macropinocytosis-related actin polymerization. Unlike KSHV-induced actin polymerization, NRP1 has a limited effect on RABV infection since NRP1 is highly expressed in RABV-non-susceptible DU145 cells (Fig. S4A and B), and Rac1, which is a key molecule for macropinocytosis, is not involved in RABV entry. Moreover, unlike NRP1, NRP2 interacts with TGFBR1 rather than TGFBR2. Whether these differences lead to RABV entry into cells via CME but KSHV entry through macropinocytosis requires further investigation. Our study strongly indicates that although some host factors or signaling pathways are shared for the entry of many viruses, the specific mechanisms may differ.

In conclusion, this study identifies NRP2 as a novel endocytic receptor of RABV that triggers actin polymerization through the TGFBR1/2-Cdc42 signaling pathway. Our findings will contribute to the understanding of RABV entry and suggest that blocking the interaction between RABV G and NRP2 would be a promising host-directed antiviral strategy.

## MATERIALS AND METHODS

### Cells and viruses

HEK293 cells (ATCC, CRL-1573) and N2a cells (ATCC, CCL-131) were maintained in DMEM containing 10% fetal bovine serum (FBS). BSR T7/5 (RRID: CVCL_RW96) cells, a BHK-derived cell line stably expressing T7 RNA polymerase (52), were obtained from the National Infrastructure of Cell Resources (Beijing, China) and maintained in DMEM containing 5% FBS. A549 cells were obtained from ATCC (ATCC, CCL-185) and maintained in Ham’s F-12K (Kaighn’s) medium (F-12K) containing 10% FBS. MDCK cells were obtained from ATCC (ATCC, PTA-6500) and maintained in DMEM containing 5% FBS. Mouse primary neuron (mPN) cells were prepared by previously established protocols (21).

RABV ERA strain, VSV, and A/WSN/1933 (H1N1 virus) were maintained in our laboratory. Recombinant ERA expressing EGFP (ERA-EGFP), in which the *EGFP* gene was inserted between the ERA *M* and *G* genes, was generated using a similar method as previously described (53). Recombinant ERA expressing G protein fused with a Flag-tag (DYKDDDDK) at the C-terminal (ERA_G/Flag_) was generated using a similar method as previously described (54). Recombinant nonreplicating E1/E3 deleted human adenovirus type 5 vectors (Ad-vector) expressing NRP2 were generated in HEK293A cells (R70507, Invitrogen) using a kit according to the instructions of the manufacturer (AdEasy Adenoviral Vector System). The viral titers were determined as the 50% tissue culture infectious dose.

### Antibodies, reagents, and chemicals

Mouse anti-NRP2 mAb (catalog number [CN]: sc-13117) for antibody blocking assay was from Santa Cruz Biotechnology. Rabbit anti-NRP2 pAb (CN: 10695-RP03, 1:200) for western blotting assay and flow cytometry assay was from Sino Biological. Mouse anti-Flag-tag pAb (CN: A00187, 1:1,000), rabbit anti-Myc-tag pAb (CN: A00172, 1:1,000), and rabbit anti-GST-tag pAb (CN: A00097, 1:1,000) were from Genscript. Mouse anti-6-His mAb (CN: 66005-1-Ig, 1:2,000) was from Proteintech. Mouse IgG2b mAb (CN: 0104-01, 1:1,000) was from Southern Biotech. Goat anti-mouse IgG coupled with Alexa Fluor 488 (CN: ab150113, 1:300) was from Abcam. Goat anti-rabbit IgG coupled with HRP (CN: 31460, 1:2,000), goat anti-mouse IgG coupled with HRP (CN: 31430, 1:2,000), and donkey anti-mouse IgG coupled with Alexa Fluor 647 (CN: A-31571, 1:1,000) were from Invitrogen. Rabbit anti-β-actin coupled with HRP mAb (CN: AC028, 1:1000) was from Abclonal. Rabbit anti-Rab5 mAb (CN: 3547S, 1:400) was from CST.

The opti-MEM (CN: 31985070), Lipofectamine RNAiMAX Transfection Reagent (CN: 13778150), 4′,6-diamidino-2-phenylindole (DAPI) (CN: D3571), Halt Protease Inhibitor Cocktail (CN: 87786), and Pierce IP Lysis Buffer (CN: 87788) were from Invitrogen. NP-40 Lysis Buffer (CN: P0013F) was from Beyotime. 1M HEPES solution (pH 7.5) (CN: SL6051) was from Coolaber. Anti-Flag-tag antibody-conjugated agarose beads (CN: A2220), cytosine β-D-arabinofuranoside (CN: V900339), and PEI transfection reagent (CN: 919012) were from Sigma-Aldrich. Glutathione Sepharose 4B beads (CN: 17-0756-01) were from GE Healthcare Bioscience. CellTiter-Glo kit (CN: G9242) was from Promega. ExFect Transfection Reagent (CN: T101-02), Phanta Super-Fidelity DNA Polymerase (CN: P501-d2), and ChamQ Universal SYBR qPCR Master Mix (CN: Q711-03) were from Vazyme. First Strand cDNA Synthesis Kit (CN: FSK-101) was from TOYOBO. AdEasy Adenoviral Vector System (CN: 240009) was from Agilent. PVDF membrane (CN: ISEQ00010) and Immobilon Crescendo Western HRP substrate (CN: WBLUR0500) were from Merck-Millipore. B27 (CN: 17504044) and TRIzol (CN: 15596018) were from Thermo Fisher Scientific. Benzonase (CN: C2001) was from HaiGene. The 4%–12% ExpressPlus PAGE Gel (CN: M41210C) and protein A resin (CN: L00210-50) were from Genscript. Superdex 200 Increase 10/300 Gl (CN: 28990944) was from Cytiva. Protein G agarose (CN: 11243233001) was from Roche. Phalloidin iFlour 488 (CN: ab176753) was from Abcam. SB431542 (CN: HY-10431), LY2109761 (CN: HY-12075), MLS-573151 (CN: HY-113849), and EHT 1864 (CN: HY-16659) were from MedChemExpress.

### Plasmids

The NRP2 full-length, NRP2 mutant, and NRP1 overexpressing plasmids were constructed by inserting the cDNA of human NRP2, NRP2 mutant, and human NRP1 into the mammalian expression vector pCAGGS with a Myc- or Flag-tag at the C-terminus. The overexpressing plasmid pTGFBR1 cyto was constructed by inserting the cDNA of the cytoplasmic domain of the human TGFBR1 into pCAGGS with a Myc-tag at the C-terminus. The overexpressing plasmid pTGFBR2 cyto was constructed by inserting the cDNA of the cytoplasmic domain of the human TGFBR2 into pCAGGS with a Myc-tag at the C-terminus. The eukaryotic expression plasmid pNRP2-Fc was constructed by inserting the cDNA of the ectodomain of human NRP2 into pcDNA3.4 with an Fc-tag at the C-terminus. The G gene of ERA, GX/09, CVS-24, and WCBV virus was respectively cloned into pCAGGS with a Myc-tag at the C-terminus. G ΔCyto was amplified by PCR from the ERA G gene using Phanta Super-Fidelity DNA Polymerase and transferred to pCAGGS with a Myc-tag at the C-terminus. G ΔCyto-mutant was synthesized by SynbioB Technology (Tianjin, China) and transferred to pCAGGS with a Myc-tag at the C-terminus.

### Eukaryotic expression and purification of soluble NRP2 ectodomain

For NRP2 ectodomain expression and purification, 300 mL HEK293F cells (A14527, Thermo Fisher Scientific) were cultured in Freestyle 293 medium in a shaker, at 37°C with 5% CO2. When the cell density reached ~2 × 10^6^ cells/mL, HEK293F cells were transfected with a plasmid encoding NRP2 ectodomain fused with Fc (NRP2-Fc) at the C-terminus using PEI transfection reagent. Cell culture supernatant was harvested at 72 h after transfection by centrifugation at 5,000 rpm for 20 min. The recombinant protein was purified using the protein A resin. The resin was washed with 30 column volumes of 20 mM Na_2_HPO_4_, 0.15 M NaCl at pH 7.0. After the wash, the protein was eluted from the resin and further purified by size-exclusion chromatography using Superdex 200 Increase 10/300 Gl. The running buffer used contains 25 mM HEPES at pH 7.0 and 150 mM NaCl. Protein purity was confirmed by SDS-PAGE analysis.

### Purification of RABV particles

BSR T7/5 cells at 80% confluence were infected with ERA_G/Flag_ virus at an MOI of 0.01. The virus-containing supernatant was harvested at 60 h post-infection and centrifuged at 8,000 rpm for 10 min at 4°C to remove the cell debris. Then the supernatant was ultracentrifuged at 15,000 rpm for 1 h in an SW 32 Ti rotor (Beckman Coulter, California, USA) at 4°C to collect the virus pellet. The pellet was dispersed in 600 µL PBS and then overlaid on top of a discontinuous sucrose density gradient (10%, 15%, 20%, 25%, and 30%). After ultracentrifugation at 18,000 rpm for 1.5 h in an SW 41 Ti rotor (Beckman Coulter) at 4°C, visible virus bands were removed by suction and diluted in PBS. The purified virus titers were detected by virus titration in BSR T7/5 cells.

### Western blotting

The plasmid-transfected cells were washed twice with cold PBS and lysed with Pierce IP Lysis Buffer containing Halt Protease Inhibitor Cocktail and Benzonase for 1 h at 4°C. Cell lysates were centrifuged at 12,000 rpm at 4°C for 10 min. The supernatants were then mixed with protein sample loading buffer and boiled for 10 min. The samples were loaded onto a 4%–12% ExpressPlus PAGE Gel and separated by electrophoresis. Proteins were transferred to a PVDF membrane. The PVDF membrane was blocked with 5% skim milk in PBS containing 0.1% Tween-20 (PBST buffer) and incubated with the indicated primary antibody at room temperature for 1 h. The membrane was then washed three times with PBS and incubated with the indicated HRP secondary antibody. After the membrane was washed three times with PBST buffer, the target protein bands were visualized with Immobilon Crescendo Western HRP substrate using the Odyssey infrared imaging system (Li-Cor BioSciences, Lincoln, NE).

### Cell viability assay

Cell viability was determined using the Cell Titer-Glo kit following the manufacturer’s instructions. In brief, cells treated with chemicals, antibodies, or soluble proteins at the indicated concentrations were maintained in opaque 96-well plates for the indicated time. Then, the medium was replaced with 100 µL of CellTiter-Glo reagent per well, and the cells were incubated for 10 min at room temperature. The luminescence was then measured using a GloMax 96 Microplate Luminometer.

### Real-time quantitative PCR (qPCR)

To quantify the level of NRP2 mRNA, NRP1 mRNA, TGFBR1 mRNA, TGFBR2 mRNA, and Rac1 mRNA, or the viral RNA (vRNA) in cells after different treatments, the total RNA from cells was extracted using TRIzol reagent. The cDNA was generated with a First Strand cDNA Synthesis Kit. The qPCR was generated with ChamQ Universal SYBR qPCR Master Mix with the specific primers listed in Table S1. qPCR was conducted using the Applied Biosystems QuantStudio 5 Real-Time PCR System (Thermo Fisher). The 2^−∆∆Ct^ method was used to calculate the relative gene level with 28S rRNA as the internal control.

### RNA interference (RNAi) assay

The RNAi assay was performed in 24-well plates. Briefly, 120 µL of opti-MEM containing 0.8 µL of Lipofectamine RNAiMAX Transfection Reagent was added to the wells in which the siRNAs were arrayed. After a 30 min incubation at room temperature, HEK293 cells (1 × 10^4^ cells/well) and N2a cells (1.2 × 10^4^ cells/well) in a volume of 380 µL were added. The siRNA-transfected HEK293 cells and N2a cells were maintained at 37°C for 60 h and 72 h, respectively, before they were infected with ERA-EGFP.

### Drug inhibition assays

For the drug inhibition assays, HEK293 cells and N2a cells were pre-treated with drugs at different concentrations for 1 h before they were infected with ERA-EGFP (MOI 0.05). At 48 h post-infection, the supernatants were harvested for virus titration in BSR T7/5 cells.

### Viral infection assay

For the RNAi assays, at 60 h post-transfection, siRNA-transfected cells were infected with ERA-EGFP (MOI 0.05) for 1 h at 37°C; for the overexpression assays, at 48 h post-transfection, the human NRP2-transfected cells were infected with ERA-EGFP (MOI 0.05) for 1 h at 37°C. The cells were then washed three times with 2% FBS-containing medium, and 2% FBS-containing medium was added. The supernatants were harvested at 24 h, 48 h, and 72 h post-infection and titrated by serial dilution in BSR T7/5 cells. Viral titers are expressed as FFU/mL.

### Viral binding assay

For the viral binding assay, the siRNA-transfected cells in 24-well plates were infected with ERA-EGFP (MOI 10) at 4°C for 1 h. The cells were then washed three times with cold PBS and lysed to measure the vRNA levels by qPCR.

### Viral internalization assay

For the viral internalization assay, the inhibitor-treated and siRNA-transfected cells in 24-well plates were infected with ERA-EGFP (MOI 10) at 4°C for 1 h. The cells were washed three times with cold PBS and maintained at 37°C for 2 h. Then, the cells were washed for 3 min with 0.25% trypsin to remove the virus retained on the surface of the plasma membrane. After that, the cells were lysed for the qPCR assay to analyze the vRNA in the cells. For the microscopy-based assay, virus-bound HEK293 cells or N2a cells were washed three times with cold PBS and maintained at 37°C for the indicated time. The cells were fixed, and the cell surface G was analyzed by performing a confocal laser scanning microscopy assay with the indicated antibodies. For the H1N1 internalization assay, the drug-treated A549 cells in six-well plates were infected with H1N1 virus (MOI 50) and incubated at 4°C for 1 h. The cells were washed three times with cold PBS and maintained at 37°C for 2 h. Then, the cells were washed eight times with cold acidic PBS (pH 1.3) to remove the virus retained on the surface of the plasma membrane. After that, the cells were lysed for the qPCR assay to analyze the vRNA in the cells.

For the VSV internalization assay, different concentrations of NRP2-Ab (2, 5, 10 µg/mL) or IgG2b- (10 µg/mL) treated HEK293 cells in 24-well plates were infected with VSV (MOI 5) at 4°C for 1 h. The cells were washed three times with cold PBS and maintained at 37°C for 1 h. Then, the cells were washed for 3 min with 0.25% trypsin to remove the virus retained on the surface of the plasma membrane. After that, the cells were lysed for the qPCR assay to analyze the vRNA in the cells. VSV (MOI 5) was mixed with different concentrations of NRP2-GST (20, 50, or 100 µg/mL) or control GST protein (100 µg/mL) in 0.3 mL of culture medium at 4°C for 1 h. HEK293 cells were infected with the protein-virus mixture at 4°C for 1 h. The cells were washed three times with cold PBS and maintained at 37°C for 1 h. Then, the cells were washed for 3 min with 0.25% trypsin to remove the virus retained on the surface of the plasma membrane. After that, the cells were lysed for the qPCR assay to analyze the vRNA in the cells.

### Multiplex immunofluorescence

N2a cells (5 × 10^4^ cells/well) were cultured on Millicell EZ slide 4-Well Glass and then infected with ERA_G/Flag_ (MOI 10) at 37°C for 20 min. The cells were thoroughly washed with PBS and fixed with 3% paraformaldehyde. Multiplex immunofluorescence with tyramide signal amplification (TSA) was performed by following the previously established protocol (21). Briefly, endogenous peroxidase activity was quenched by 0.3% hydrogen peroxide in PBS for 20 min at room temperature. After permeabilization with 0.1% Triton X-100 and blocking steps (Zsbio, ZLI-9056, Beijing, China), samples were incubated with primary antibody at 4°C overnight, followed by HRP-conjugated secondary antibody at room temperature for 30 min. TSA amplification reagent diluted 1:100 in reaction buffer (NEL811001 KT, PerkinElmer, Waltham, MA, USA) was added to the sample and incubated for 30–120 sec until the best signal intensity and signal-to-noise ratio were achieved. The primary and secondary antibodies were then removed by incubation with stripping buffer at 37°C for 30 min while retaining the TSA signal. After a brief rinse, other antigens were serially detected using spectrally different TSA reagents following the above method. The primary antibodies used in this study were mouse anti-NRP2 mAb, mouse anti-Flag pAb, and rabbit anti-Rab5 mAb. The secondary antibodies were goat anti-rabbit IgG coupled with HRP and goat anti-mouse IgG coupled with HRP. Images were collected with a Z-stack size of 0.25 µm using a Zeiss LSM880 laser-scanning confocal microscope equipped with Airyscan. Cells were scanned 36 layers along the Z axis with a pixel dwell time of 1 microsecond. The resolution of the acquired images was 1904 × 1904.

Data were processed using Bitplane Imaris software (Bitplane AG, Zurich, Switzerland) by following the established protocol (21). Briefly, first, the green channel (RABV) and purple channel (Rab5) were processed using the “surface module.” Then, the surface results of the purple and green channels were inputted as “cell” and “nuclei,” respectively, under the “cell module.” Next, the red channel (NRP2) was processed using the “surface module.” After that, a new channel was established by merging the green and red channels using the “mask data set” of the “coloc” module. Spots that represented the co-localization of RABV and NRP2 were counted in the merged channel by using the “spot” module. Finally, the spot results were inputted into “cell,” and the RABV-NRP2 spots that co-localized with Rab5 were counted.

### Co-immunoprecipitation

For the co-immunoprecipitation assay, the treated cells were lysed as described for the western blotting assay, and the supernatants of the cell lysates were mixed with 40 µL of protein G Agarose for 4 h at 4°C on a flip shaker. The protein G beads were removed by centrifugation, and the supernatants were collected and mixed with anti-Flag-tag antibody-conjugated agarose beads for 12 h at 4°C on the flip shaker. After conjugation, the beads were washed five times with cold NP-40 Lysis Buffer. The beads were resuspended in PBS and mixed with protein sample loading buffer and boiled for 10 min, then subjected to SDS-PAGE and assessed by western blot analysis.

### Pull-down assay

The N-terminal GST-tagged soluble ectodomain of NRP2 (NRP2-GST, amino acids [aa] 17–865) and ERA G-His (aa 41–450) proteins were expressed in *E. coli* and purified by FriendBio Technology (Wuhan, Hubei, China). GST protein was used as the negative control. The purified GST-tagged proteins were, respectively, incubated with Glutathione Sepharose 4B beads at 4°C for 2 h. The beads were then washed with cold NP-40 Lysis Buffer and incubated with ERA G-His or whole-cell lysates from HEK293 cells expressing Flag-tagged or Myc-tagged proteins at 4°C for 12 h with constant rotation on a flip shaker. After conjugation, the beads were washed five times with wash buffer (pH 8.5, 20 mM Tris, 500 mM NaCl, and 2 mM EDTA) and re-suspended in PBS and mixed with protein sample loading buffer and boiled for 10 min. They were then subjected to SDS-PAGE and assessed by western blot analysis. To detect the interaction between NRP2-Fc and ERA G, the anti-Flag agarose beads were incubated with whole-cell lysates from HEK293 cells expressing Flag-tagged ERA G at 4°C for 12 h with constant rotation on a flip shaker. After conjugation, the beads were incubated with eukaryotic purified NRP2-Fc at 4°C for 12 h on the flip shaker and washed five times with wash buffer (pH 8.5, 20 mM Tris, 500 mM NaCl, and 2 mM EDTA). Then they were subjected to SDS-PAGE and assessed by western blot analysis as described above.

To detect the interaction between NRP2 and ERA_G/Flag_ particles, 5 µg Fc or 5 µg NRP2-Fc was mixed with 50 µL protein G beads at 4°C for 2 h, respectively. After conjugation, the beads were mixed with different amounts of purified ERA_G/Flag_ particles (10, 20, or 50 µL, FFU/mL = 10^8.2^) in 0.2 mL of PBS. After 2 h, the samples were washed eight times with PBS. Then, they were assessed by western blot analysis and qPCR analysis using RABV-specific primers to detect the captured vRNA, respectively.

### Protein interaction competition assay

3 µg NRP2-Fc protein was pooled with 6 µg NRP2-Ab or 6 µg IgG2b for 1 h at 4°C, respectively. Then the mixture was incubated with protein G agarose beads for 2 h at 4°C. After conjugation, the samples were washed three times with PBS, then incubated with purified ERA_G/Flag_ particles (50 µL, FFU/mL = 10^8.2^) in 0.2 mL of PBS. After 2 h, the samples were washed five times with PBS. Then they were subjected to SDS-PAGE and western blot as described above.

Purified ERA_G/Flag_ particles (50 µL, FFU/mL = 10^8.2^) were pooled with 10 µg NRP2-GST or 10 µg GST for 1 h at 4°C, respectively. Then they were mixed with NRP2-Fc-conjugated protein G agarose beads in 0.2 mL of PBS. After 2 h, the samples were washed five times with PBS. Then they were subjected to SDS-PAGE and western blot as described above.

### Antibody blocking assay

A monoclonal antibody against NRP2 (NRP2-Ab) was used for the antibody blocking assay. HEK293 cells, N2a cells, or mPN cells were incubated with 0.1 mL of culture medium containing different concentrations of NRP2-Ab (2, 5, 10, or 15 µg/mL) or IgG2b (15 µg/mL) at 4°C for 1 h. After three washes with culture medium containing the corresponding antibodies. The cells were then infected with the antibody-virus mixture and incubated at 4°C for 1 h. After another round of three washes, the cells were incubated with 0.1 mL of culture medium containing the corresponding antibodies at 37°C. At 48 h post-infection, the supernatants were collected for virus titration in BSR T7/5 cells.

### Soluble NRP2 ectodomain neutralization assay

The soluble NRP2 ectodomain neutralization assay was performed on HEK293 cells, N2a cells, or mPN cells with the soluble protein of NRP2 (NRP2-GST) that was expressed and purified by FriendBio Technology (Wuhan, Hubei, China). Viruses were mixed with different concentrations of NRP2-GST (20, 50, or 100 µg/mL) or control GST protein (100 µg/mL) in 0.3 mL of culture medium at 4°C for 1 h. HEK293 cells, N2a cells, or mPN cells in 96-well plates were infected with the protein-virus mixture and incubated at 37°C for 1 h. The cells were washed three times with culture medium to remove the unbound viruses, and the cells were then maintained with 0.1 mL of culture medium containing the corresponding proteins at 37°C for 48 h. The supernatants were collected for virus titration in BSR T7/5 cells.

### Flow cytometry

HEK293 cells were infected with ERA (MOI 5) at 37 ˚C for 1 h. Cells were scraped from the plate using cell scrapers, filtered through a 35 µm nylon mesh filter, and then collected in a 1.5 mL tube. Then, the cells were washed three times with FACS wash buffer (PBS containing 2% FCS) and fixed with 3% paraformaldehyde at room temperature for 15 min. The fixed cells were washed three times and incubated with mouse anti-NRP2 mAb as the primary antibody and goat anti-mouse IgG coupled with Alexa Fluor 488 as the secondary antibody. To detect the expression of NRP1 or NRP2, DU-145 cells, DU-145-Ad-NRP2 cells, or DU-145-Ad-vector cells were seeded onto 6-well plates for 16 h, then treated as described above. All cells were analyzed using an FC500 flow cytometer (Beckman Coulter). Cell surface fluorescence density was measured and analyzed by using FlowJo software. Briefly, the isotype control was first used to define the gating strategy to establish a baseline for nonspecific binding and background fluorescence; subsequently, the defined gates were applied to the samples to identify and quantify NRP2-expressing cell populations.

### Immunoelectron microscopy localization of NRP2 and RABV

NRP2-Myc-overexpressing N2a cells were incubated with RABV (MOI 200) at 4°C for 1 h; the bound virus was allowed to internalize at room temperature for 2 min post-binding. The cells were then fixed with 1% glutaraldehyde and 4% PFA and incubated at 4°C for 2 h. The samples were dehydrated with 50%, 70%, 90%, and 100% DMF, respectively, at 4°C for 15 min. Then, the samples were processed for embedding with UV irradiation polymerization at −20°C for 10 days. Then, the samples were incubated with the primary antibody of Myc-tag for 1 h at room temperature. After several washes with distilled water, the samples were incubated with the secondary antibody of goat anti-rabbit IgG coupled with gold (10 nm) for 40 min at room temperature. After abundant washes with distilled water, the samples were stained with uranyl acetate for 10 min, and images of the localization of NRP2 and RABV were acquired using Hitachi-7650 transmission electron microscopy.

### F-actin polymerization assay

For the F-actin polymerization assay, siControl- or siNRP2-transfected HEK293 cells were incubated with or without ERA (MOI 10) at 37°C for 30 min, and the drug-treated HEK293 cells were infected with ERA (MOI 10) at 37°C for 30 min; the cells were fixed and washed three times with PBS and incubated with Phalloidin iFlour 488 for 1 h. Then, the F-actin polymerization in the cells was analyzed by performing a confocal laser scanning microscopy assay.

## Data Availability

The data supporting the findings of this study are available within the article and its supplemental material.

## SUPPLEMENTAL MATERIAL

Supplemental material jvi.00638-25-s0001.docx Fig. S1 to S4; Table S1.

ASM does not own the copyrights to Supplemental Material that may be linked to, or accessed through, an article. The authors have granted ASM a non-exclusive, world-wide license to publish the Supplemental Material files. Please contact the corresponding author directly for reuse.
