# Supplementary material for: Rabies virus utilizes neuropilin 2 as an endocytic receptor to trigger TGFBR1-mediated actin polymerization
Source: J Virol. 2025 Jun 25;99(7):e00638-25. doi: 10.1128/jvi.00638-25 (PMC12282144; doi:10.1128/jvi.00638-25)
Supplement: Supplemental material — Fig. S1 to S4; Table S1. [file jvi.00638-25-s0001.docx]

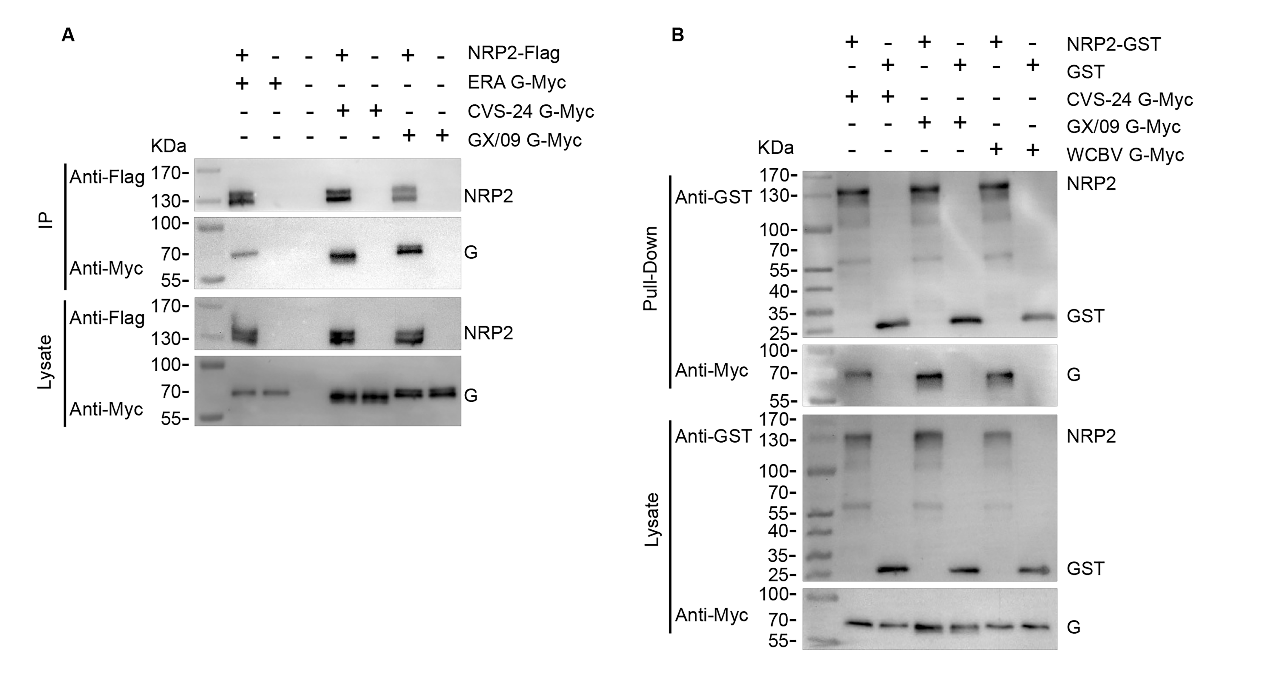
 **Fig S1. NRP2 interacts with ERA G, CVS-24 G, GX/09 G, and WCBV G.** (A) HEK293 cells were co-transfected with NRP2-Flag and ERA G-Myc, CVS-24 G-Myc, or GX/09 G-Myc, and then subjected to immunoprecipitation (IP) by using anti-Flag agarose beads. Representative western blots of whole-cell lysates and eluates after IP are shown. (B) Purified NRP2-GST protein was pooled with the lysate from CVS-24 G-Myc-, GX/09 G-Myc-, or WCBV G-Myc-transfected HEK293 cells and then pulled down using anti-GST beads. The GST protein was used as the negative control.


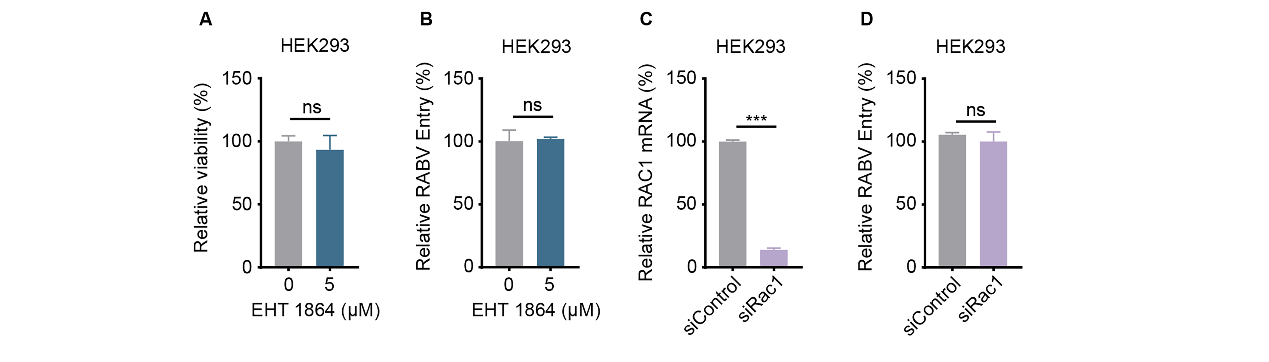
**Fig S2. Rac1 is not required for RABV entry.** (A) HEK293 cells were treated with EHT 1864 at the indicated concentration for 48 h at 37°C, and then cell viability was determined by using the Cell Titer Glo kit. (B) RABV internalization was quantified in EHT 1864-treated HEK293 cells by qPCR and the results were normalized to DMSO-treated cells. (C) The Rac1 mRNA level in the indicated siRNA-transfected HEK293 cells was measured by qPCR. siRac1, siRNA specific for Rac1 mRNA. (D) RABV internalization was quantified in Rac1-silenced HEK293 cells by qPCR and the results were normalized to the scrambled siRNA-transfected cells. The data shown in panels (A to D) are means ± SD of three independent experiments. Statistical analysis was performed by using the unpaired, two-tailed Student’s test, ns, not significant, ****P*<0.001.


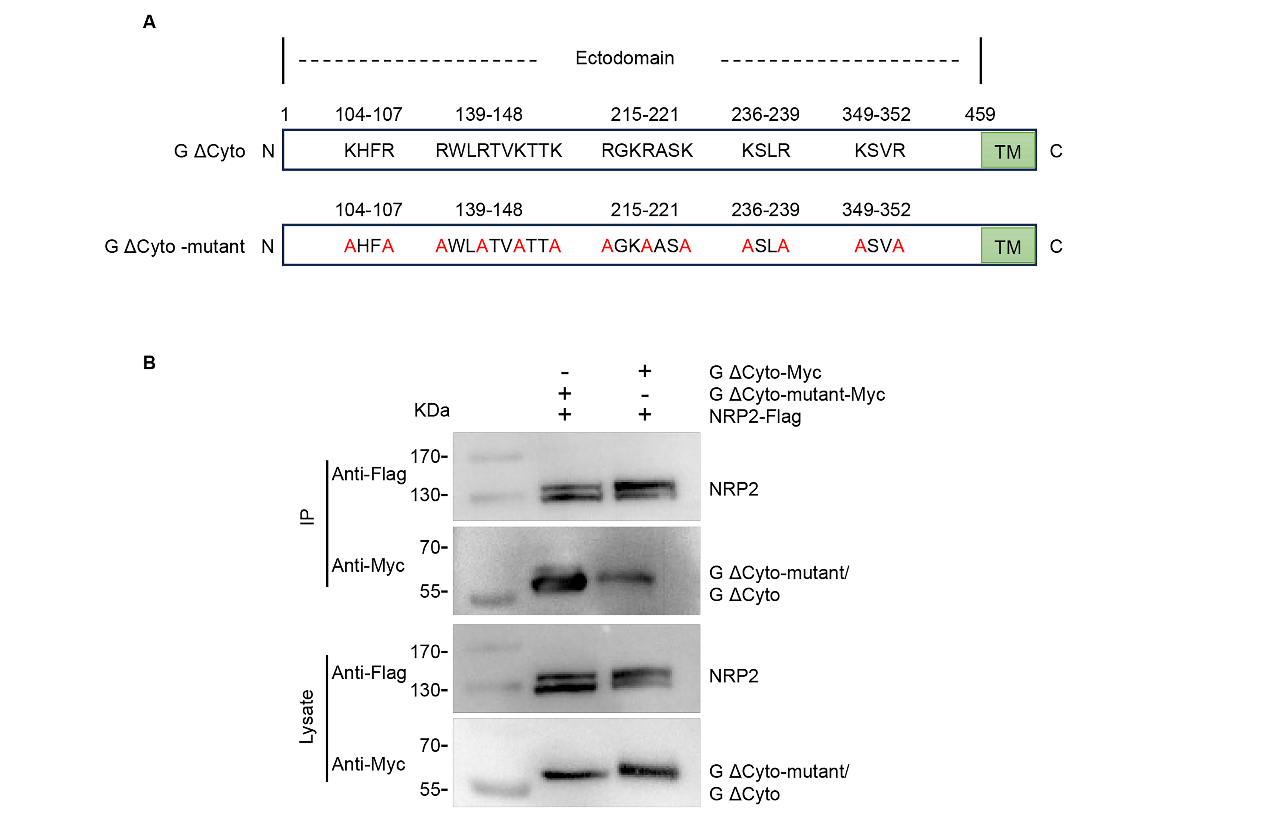
 **Fig S3. RABV G binding to NRP2 is independent of its CendR motifs.** (A) Schematic representation of CendR motifs mutation sites in the ectodomain of RABV G. (B) The interaction between NRP2 and G ΔCyto or G ΔCyto-mutant was analyzed by using co-immunoprecipitation with anti-Flag agarose beads.


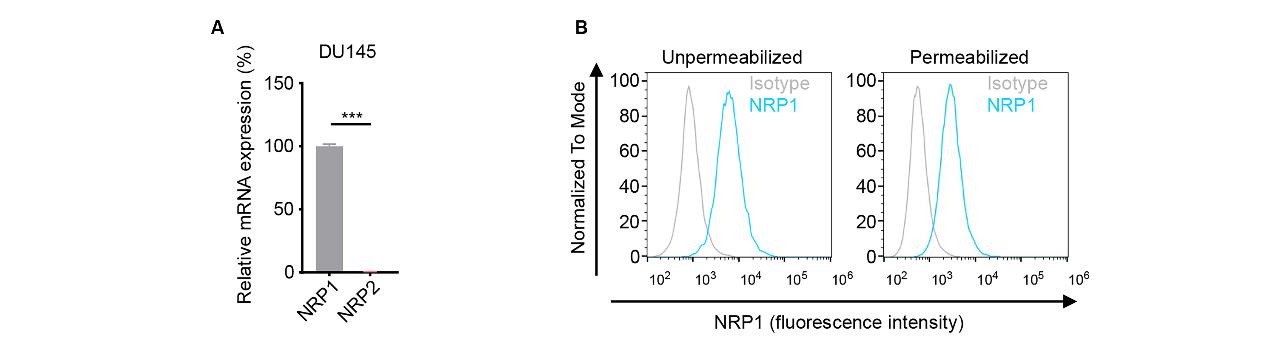


**Fig S4. NRP1 is expressed in DU145 cells.** (A) The NRP1 mRNA and NRP2 mRNA levels in DU145 cells were measured by qPCR. (B) The expression of NRP1 was detected by flow cytometry under unpermeabilized or permeabilized conditions in DU145 cells. The data shown in panel (A) are means ± SD of three independent experiments. Statistical analysis was performed by using the unpaired, two-tailed Student’s test, ****P<0.001*.

**Table S1. Sequence information for the oligoes used in this study.**

| Gene | Catalog number | Forward sequence (5'-3') | Reverse sequence (5'-3') | Application |
| --- | --- | --- | --- | --- |
| 28S rRNA | N/A | GGGTGGTAAACTCCATCTAAGG | GCCCTCTTGAACTCTCTCTTC | qPCR assay |
| RABV-N | N/A | ATGAAGACTGTTCAGGACTGGTAT | CCCTGGCTCAAACATTCTTCTTA | qPCR assay |
| VSV-P | N/A | GTGACGGACGAATGTCTCATAA | TTTGACTCTCGCCTGATTGTAC | qPCR assay |
| H1N1-HA | N/A | CAGTCACAATAGGAGAGTGCCC | CCATCCCCCCTCAATAAAAC | qPCR assay |
| NRP2 (human) | N/A | TCAAGACAGGCTCTGAAGATTG | CAGTCCAAGTTGTGTGGATACT | qPCR assay |
| NRP2 (mouse) | N/A | CCAGGGTTTCCAGAGAAGTATC | GTCAAAGGTCAGGAACTGTAGG | qPCR assay |
| NRP1 | N/A | GGGAAGACTGGATCACCATAAA | GGAATACTGCAACCACAACATC | qPCR assay |
| TGFBR1 | N/A | GATCTTGTACCTTCTGACCCATC | TCTGCCATCTGTTTGGGATATT | qPCR assay |
| TGFBR2 | N/A | CAACGGTGCAGTCAAGTTTC | CTTCTCACAGATGGAGGTGATG | qPCR assay |
| Rac1 | N/A | GGATACAGCTGGACAAGAAGAT | CAAATGATGCAGGACTCACAAG | qPCR assay |
| NRP2 (human) | SASI_Hs01_00172646 | GCAAGUAUGACUUUAUCGAdTdT | UCGAUAAAGUCAUACUUGCdTdT | RNAi assay |
| NRP2 (mouse) | SASI_Mm02_00292582 | AUUGUUCGCUUGGAAUAUCdTdT | GAUAUUCCAAGCGAACAAUdTdT | RNAi assay |
| TGFBR1 | SASI_Hs01_00181142 | CUUACAGCAUUGCGGAUUAdTdT | UAAUCCGCAAUGCUGUAAGdTdT | RNAi assay |
| TGFBR2 | SASI_Hs01_00099216 | GAAAUGACAUCUCGCUGUAdTdT | UACAGCGAGAUGUCAUUUCdTdT | RNAi assay |
| Rac1 | SASI_Hs01_00015565 | GCAAACAGAUGUGUUCUUAdTdT | UAAGAACACAUCUGUUUGCdTdT | RNAi assay |
